# Croaker Fish Bone-Derived Hydroxyapatite as a Sustainable Source for 3D-Printed Scaffolds for Bone Regeneration

**DOI:** 10.3390/md24080260

**Published:** 2026-07-26

**Authors:** Diana Gabriela Nina-Nina, Giovanna de Amorim Grasser, Amanda Sardeli Alqualo, João Paulo dos Santos Prado, Eliandra de Sousa Trichês, Elson Longo, Ana Cláudia Muniz Rennó, Anna Rafaela Cavalcante Braga, Marcelo Assis, Renata Neves Granito

**Affiliations:** 1 Department of Biosciences, Universidade Federal de São Paulo (UNIFESP), Santos 11015-020, Brazil; dnina@unifesp.br (D.G.N.-N.); amanda.assaf@unifesp.br (A.S.A.); paulo.prado@unifesp.br (J.P.d.S.P.); a.renno@unifesp.br (A.C.M.R.); anna.braga@unifesp.br (A.R.C.B.); 2 Center for the Development of Functional Materials (CDMF), Universidade Federal de São Carlos (UFSCar), São Carlos 13565-905, Brazil; giovanna.grasser@estudante.ufscar.br (G.d.A.G.); elson.liec@gmail.com (E.L.); 3 Institute of Science and Technology, Universidade Federal de São Paulo (UNIFESP), São José dos Campos 12231-280, Brazil; eliandra.sousa@unifesp.br; 4 Department of Chemical Engineering, Universidade Federal de São Paulo (UNIFESP), Diadema 09972-270, Brazil; 5 Institute for Advanced Marine Studies (IEAMar), São Paulo State University (UNESP), São Vicente 11350-011, Brazil

**Keywords:** hydroxyapatite, croaker fish bone, bioactive scaffolds, bone tissue engineering

## Abstract

The use of biogenic hydroxyapatite as a sustainable and bioactive alternative to synthetic ceramics has attracted increasing attention for 3D-printed scaffolds in bone tissue engineering. In this work, calcium alginate-based scaffolds reinforced with commercial (cHA) and biogenic hydroxyapatite (bHA) obtained from croaker fish bones (*Micropogonias furnieri*) were fabricated by 3D printing using hydroxyapatite contents ranging from 10% to 20%. Both hydroxyapatites exhibited hexagonal structures, and all formulations showed rheological behavior suitable for extrusion-based printing. Structural analyses revealed increased diffraction peak intensity with higher hydroxyapatite content, while FTIR spectra showed no significant structural changes. Hydroxyapatite addition increased the compressive modulus, although higher loadings reduced maximum resistance and produced denser, less porous structures. After 14 days in simulated body fluid, scaffolds containing 10% bHA favored apatite deposition, evidenced by increased phosphorus levels. In vitro assays using MC3T3-E1 pre-osteoblasts demonstrated biocompatibility, with metabolic viability above 70% and no toxicity. The 10% bHA formulation also enhanced cell proliferation, adhesion, and migration without increasing reactive oxygen or nitrogen species. Alizarin Red staining indicated osteogenic potential, while micronucleus assays with CHO-K1 cells confirmed the absence of genotoxicity. These findings highlight the potential of biogenic hydroxyapatite scaffolds for bone tissue engineering.

## 1. Introduction

Bone fractures and disorders associated with them have become an increasingly relevant public health concern, since they are commonly linked to loss of functional capacity, temporary or permanent work restrictions, reduced productivity, deterioration of quality of life, and a marked rise in healthcare expenditures [1,2]. In 2021, approximately 172.79 million new bone fracture cases were reported worldwide, while the number of prevalent cases reached 453.31 million [3]. Furthermore, these figures reflect a substantial increase over the past three decades, highlighting the growing global burden associated with bone fractures [3]. In this challenging scenario, bone tissue engineering has dedicated efforts to the development of bone substitutes capable of repairing or replacing damaged bone tissue regions in a functional and biologically compatible manner [4].

For bone defect repair to be successful, the selected substitutes must balance structural, chemical, and biological characteristics to enable safe interaction with the physiological environment [5]. In addition to biocompatibility and biodegradability, it is desirable that they allow adjustments to specific clinical needs and are viable from a production standpoint [6]. From a functional standpoint, bone substitutes must do more than just exist in the body; they must behave like bone. This involves presenting a chemical composition close to that of natural bone, mechanical strength within a compatible range, and a 3D structure that breaks down gradually enough for the surrounding tissue to replace it progressively [7,8]. Choosing the proper bone substitute requires more than just biocompatibility; the material must integrate structural integrity with chemical stability to function safely in the body [9,10,11].

Hydroxyapatite has occupied a leading position in bone tissue engineering due to its chemical composition, which closely resembles the mineral phase of the bone matrix [12,13]. The interest in hydroxyapatite-based materials stems from their ability to bond and integrate with native bone tissue. These ceramic materials not only serve as passive structural supports but also exhibit osteoconductive properties that favor the deposition of the mineral matrix due to their chemical similarity to the inorganic phase of bone [14,15]. Beyond these properties, its slow degradation contributes to maintaining structural support during the initial repair phases [16]. Despite these advantages, the low resistance of hydroxyapatite-based ceramic materials limits their application [17].

In line with the Ocean Decade, this ecosystem has increasingly been recognized as a relevant source of natural bioactives with high added value, which has stimulated interest in the recovery and reuse of residues and by-products generated, especially by the fishing supply chain [18]. Beyond performance, these materials can be a sustainable pathway to reduce the carbon footprint and position technology development within the circular economy. In this scenario, low-cost alternatives for producing hydroxyapatite from waste generated by the fishing industry can be attractive compared to conventional chemical routes [19]. In the fish industry, meat represents the main commercial product, while skin, scales, and bones are frequently discarded or destined for low-value uses. However, these by-products have high potential for valorization: skin and scales can be processed for the extraction of collagen [20], which is widely used in the food, cosmetic, and pharmaceutical sectors, while bones and scales themselves constitute promising sources of hydroxyapatite for biomaterial applications [21,22]. Xavier et al. observed that the production of hydroxyapatite from fish bones can generate new economic opportunities, stimulate the creation of companies and jobs, and contribute to the reduction in waste, especially in a scenario favorable to innovation and sustainability policies [23].

In recent years, several authors have investigated the potential of hydroxyapatite derived from fish bones as a sustainable and biologically compatible alternative for application in bone tissue engineering. In the study conducted by Shi et al., different natural hydroxyapatites obtained from rainbow trout (*Oncorhynchus mykiss*), cod (*Gadus* sp.), and salmon (*Oncorhynchus keta*) bones were compared, showing better biological compatibility than synthetic hydroxyapatite, an effect attributed to the presence of carbonates and magnesium in their composition [24]. Similar results were reported by Venkatesan et al., who extracted nano-hydroxyapatite from salmon bones by alkaline hydrolysis and verified the absence of toxicity and increased mineralization in mesenchymal stem cells, reinforcing the material’s potential as an osteogenic biomaterial [25]. Other studies highlight the production of hydroxyapatite from the bones of different fish species, including swordfish (*Xiphias gladius*), tuna (*Thunnus thynnus* and *Katsuwonus pelamis*), sardine (*Sardinella longiceps*), sea bass (*Lates calcarifer*), and red bigeye (*Priacanthus macracanthus*), generally showing good biocompatibility and promising performance for applications in bone tissue engineering [26,27,28,29,30,31]. Nam et al. emphasize that the morphology, surface area, and particle size of hydroxyapatite vary across species, which can influence its performance in biomedical applications [32].

Taken together, these findings demonstrate that fish-derived hydroxyapatite not only represents a sustainable strategy for the valorization of fish-processing residues, but also offers physicochemical and biological characteristics that may enhance bone regeneration. In this context, the investigation of new biogenic sources becomes particularly relevant, especially when considering regional availability and the possibility of obtaining biomaterials with distinct biological properties. This work evaluated the potential use of hydroxyapatite obtained from croaker fish bones (*Micropogonias furnieri*), a widely commercialized fish on the southern coast of Brazil, for applications in bone tissue engineering.

## 2. Results and Discussion

### 2.1. Extraction of Biogenic Hydroxyapatite from Fish Bones and Physicochemical Characterization

**Figure 1:**
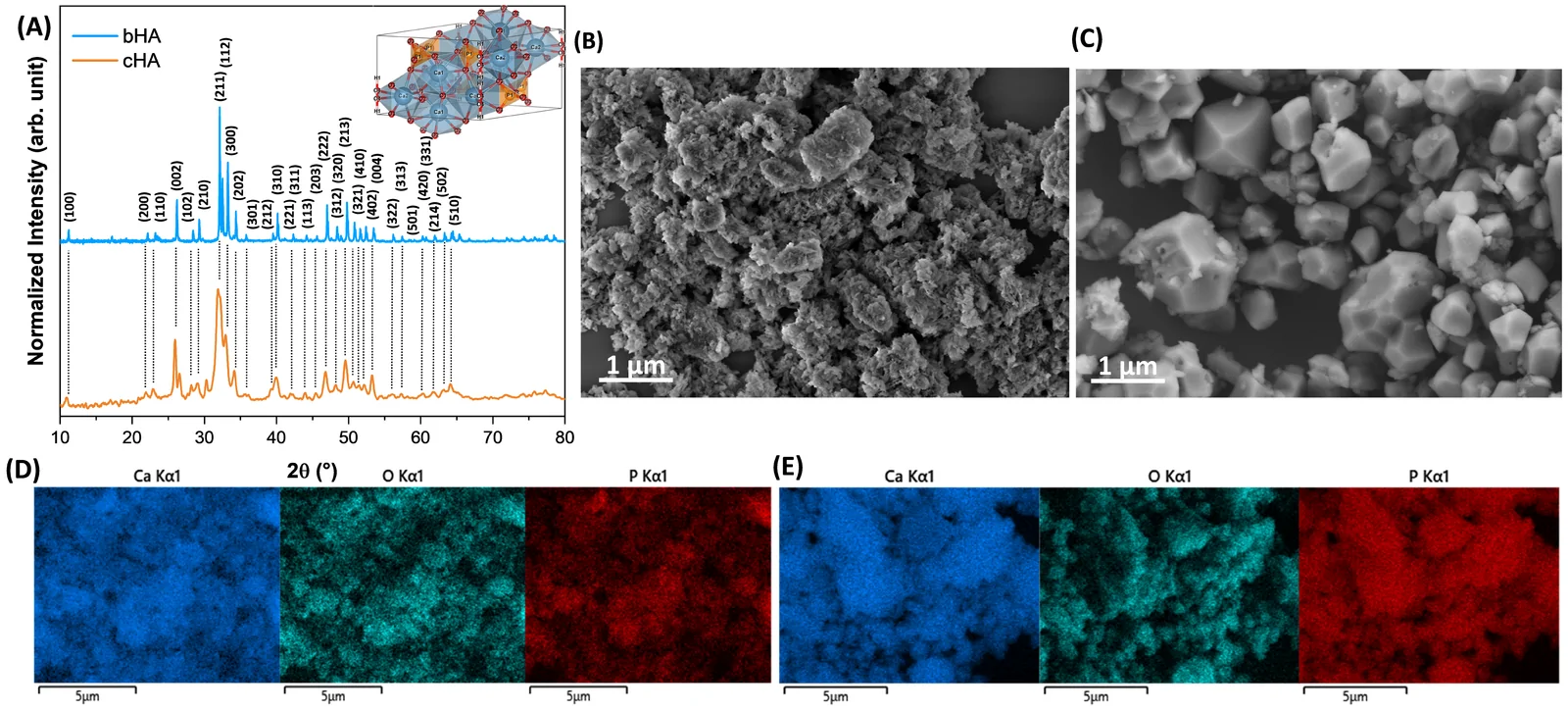
(**A**) XRD of hidroxiapatite samples. SEM images of (**B**) cHA and (**C**) bHA. EDS mapping Ca, P and O for samples (**D**) cHA and (**E**) bHA.

The structures of both cHA and bHA were initially analyzed by XRD to evaluate their phases and crystallinity (Figure 1A). In both cases, the results confirm hexagonal hydroxyapatite phase Ca_10_(PO_4_)_6_(OH)_2_ with space group P 63/m (ICSD No. 169498) [33], which is composed of [CaO_9_], [CaO_8_H_2_], and [PO_4_] complex clusters. While both materials were obtained as single-phase hydroxyapatite, they exhibited marked differences in crystallinity. The cHA displayed broad diffraction peaks, indicating a lower degree of crystalline order, whereas the bHA exhibited sharp, well-defined reflections, consistent with a more ordered crystal structure. The crystallinity index (CI), calculated according to the method proposed by Landi et al., was 35.0% for cHA and 80.2% for bHA, quantitatively confirming the substantially higher crystallinity of the biogenic hydroxyapatite [34]. This higher crystalline order is attributed to the calcination step performed at 800 °C during the preparation of bHA, which promotes crystal growth and reduces structural disorder.

The morphology of both hydroxyapatite samples was analyzed by SEM (Figure 1B,C). The cHA morphology consists of microagglomerates formed by nanometer-sized hydroxyapatite rods. Machado et al. observed a similar morphology in their study, in which the hydroxyapatite was obtained by a controlled precipitation method [35]. In contrast, the bHA exhibits a morphology of irregular polyhedra, which is a result of the top-down milling process used in this study. Particle size distribution was obtained by granulometry and showed that for the cHA before milling, the microagglomerates measured 10.39 µm, decreasing to 6.29 µm afterward. For bHA, the size decreased from 39.12 µm before milling to 2.40 µm after milling. Elemental composition was obtained by EDS (Figure 1D,E). Both samples contain Ca, P, and O; however, their Ca/P ratios differ. While the theoretical Ca/P ratio of hydroxyapatite is 1.67, the values obtained for cHA and bHA were 1.56 and 1.96, respectively. These results show that the cHA composition is very close to the nominal value, whereas the bHA exhibited a higher Ca/P ratio. Since this ratio was obtained by semiquantitative EDS analysis and no secondary crystalline phases were detected by XRD, the observed difference is attributed to the intrinsic compositional variability of the biogenic hydroxyapatite [36].

### 2.2. Rheology

**Figure 2:**
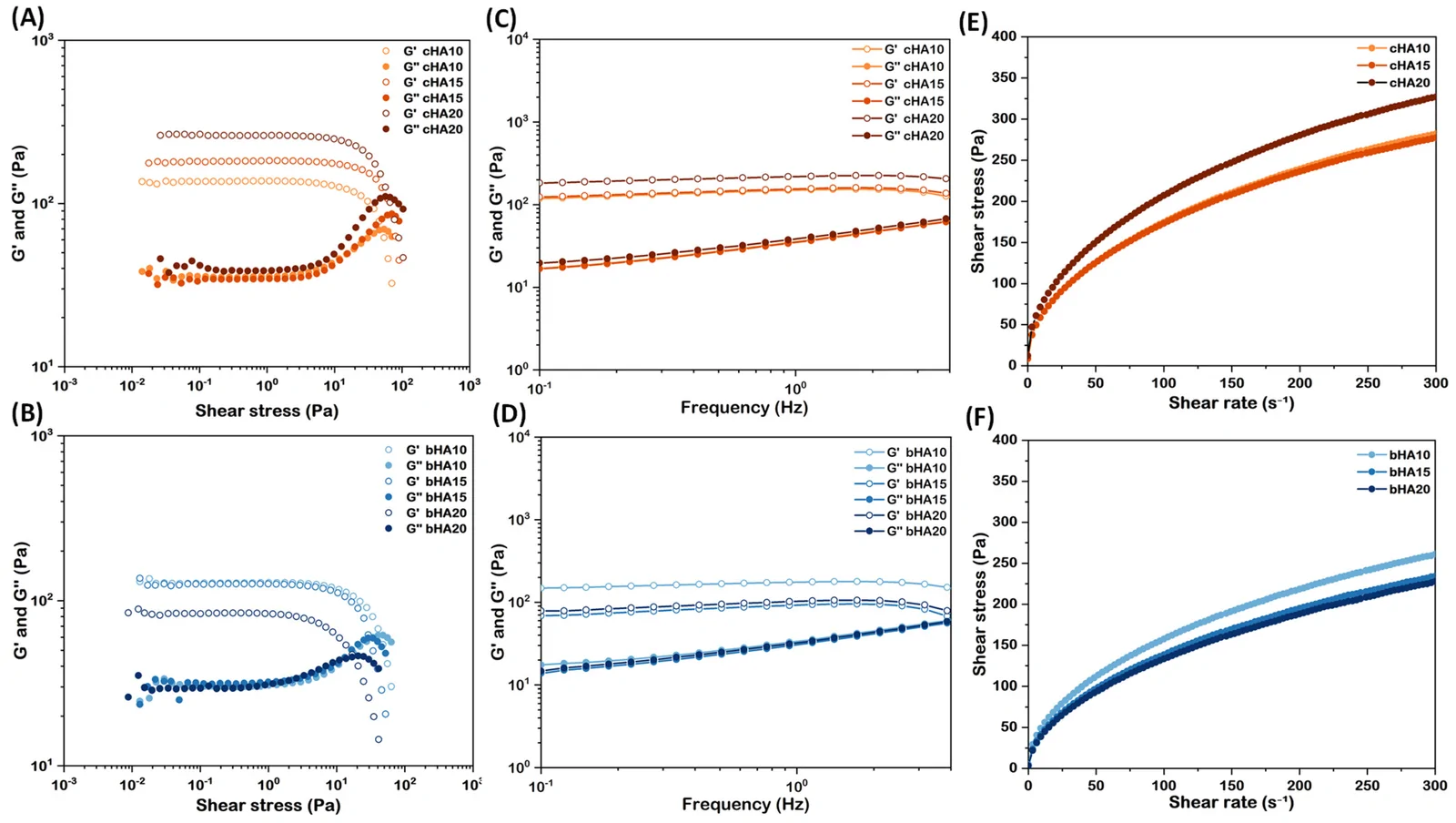
Rheological properties of cHA and bHA inks: (**A**,**B**) G′ and G″ vs. shear stress; (**C**,**D**) G′ and G″ vs. frequency; (**E**,**F**) Flow curves.

After the characterization of cHA and bHA, they were incorporated at 10, 15 and 20% into a sodium alginate solution and subsequently cross-linked with Ca^2+^ to form Ca-alginate. The viscoelastic properties of these ink formulations were assessed through oscillatory shear stress sweeps (Figure 2A,B), since these properties are critical to stability and reproducibility during the 3D printing process. In all cases, the storage modulus G′ remained consistently higher than the loss modulus G″, indicating dominant elastic behavior within the LVR. Among the cHA-based samples, the formulation with the highest concentration (20%, cHA20) exhibited the highest G′ values, whereas cHA10 (10%) showed the lowest. For the bHA samples, the opposite trend was observed: bHA10 (10%) and bHA15 (15%) showed the highest G′, while bHA20 (20%) showed lower values. The cHA-loaded inks demonstrated higher G′ and G″ than their bHA counterparts, which can be attributed to differences in the size and morphology of the hydroxyapatite particles. The high aspect ratio of the cHA nanorods may promote a more robust percolated network, thereby enhancing the rheological stiffness [37,38]. For the cHA samples, a crossover point between G′ and G″ occurred at a shear stress of approximately 60 Pa, in contrast to the bHA samples, where a shift in crossover values from 60 Pa down to 15 Pa occurred at lower bHA concentrations. Beyond these shear stress thresholds, G′ began to decline as G″ increased, marking the onset of structural breakdown.

From the previous results of the stress sweeps, a constant shear stress of 1 Pa was established for the frequency analysis (Figure 2C,D). For all samples, G′ consistently exceeded G″, reinforcing the conclusion that these materials behave predominantly as elastic solids. Furthermore, G″ exhibited a slight frequency dependence, increasing with frequency, a typical rheological signature of well-structured hydrogel networks. The flow curves for all formulations revealed a distinctive third stage characterized by a gradual rise in shear stress at higher shear rates (Figure 2E,F). This behavior highlights the material’s complex thixotropic recovery, where the disrupted gel network begins to reorganize and resist flow even under continued deformation [39]. In the context of 3D printing, this suggests a rapid structural stabilization of the ink immediately after leaving the nozzle, a critical factor for maintaining the shape fidelity of the printed layers.

### 2.3. X-Ray Diffraction (XRD)

**Figure 3:**
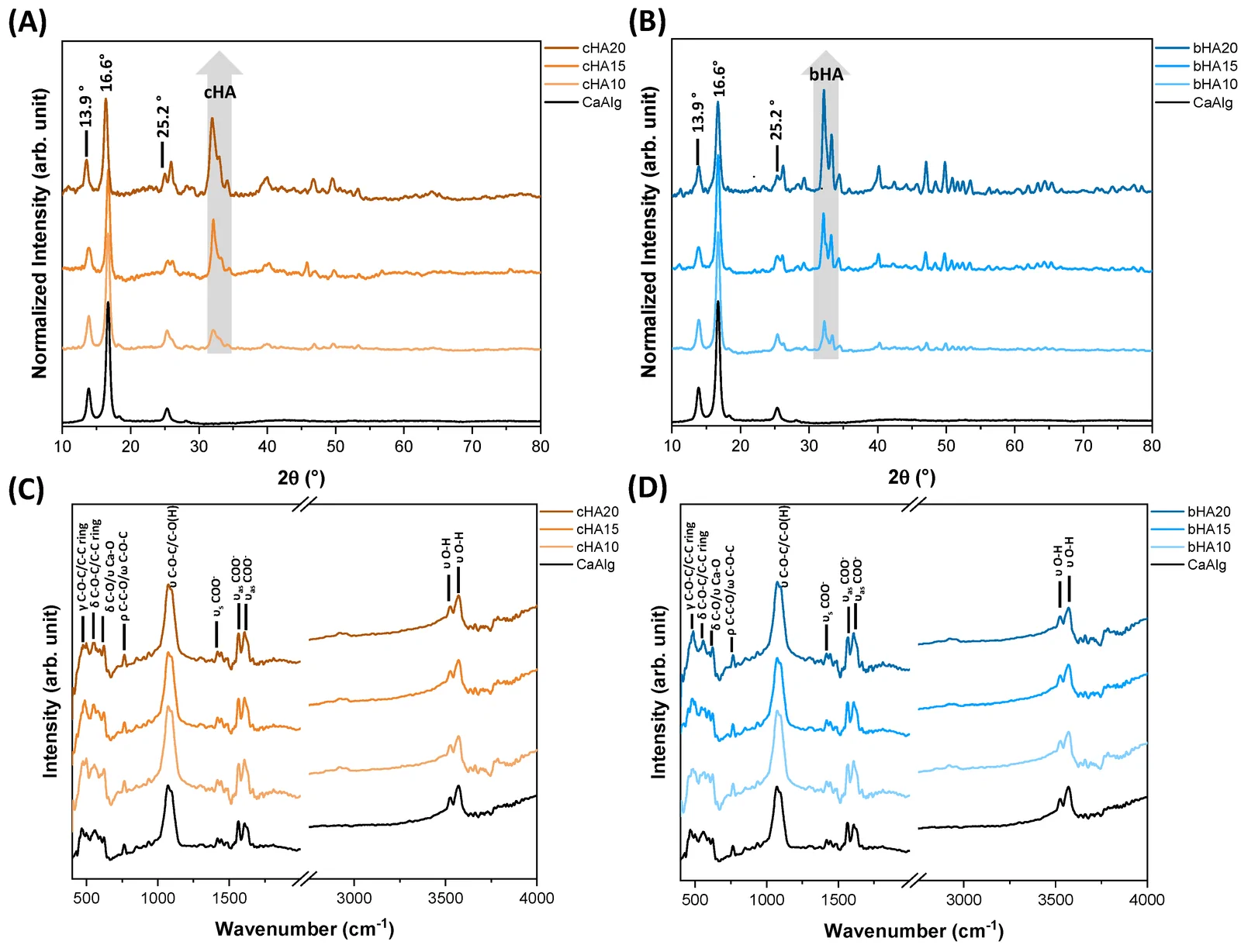
X-ray diffractograms for scaffolds using (**A**) cHA and (**B**) bHA. FTIR spectra for scaffolds using (**C**) cHA and (**D**) bHA.

After 3D printing, the scaffold structure was analyzed by XRD. XRD results (Figure 3A,B) show that the alginate scaffolds crosslinked with Ca^2+^ possess crystalline domains due to the formation of the egg-box structure. The peak located at 13.9° is related to the crystalline phase associated with mannuronate-rich (M-block) segments, while the higher intensity peak located at 16.6° corresponds to the highly ordered guluronate domains (G-blocks) coordinated with Ca^2+^ in egg-box-type arrangements [40,41]. A third peak located at 25.2° is observed, which is related to crystalline short-range chain packing of the Ca-alginate network [41]. Regarding the composites, the addition of either cHA or bHA to the scaffolds results in the appearance of peaks characteristic of their hexagonal structure. In both instances, peak intensity scales with the amount of incorporated material, while overall crystallinity is preserved. As expected, the cHA produces less defined peaks, whereas the bHA produces well-defined peaks.

### 2.4. Fourier Transform Infrared Spectroscopy (FTIR)

FTIR analysis (Figure 3C,D) reveals no significant differences between scaffolds prepared with bHA versus cHA. All spectra primarily display vibrations characteristic of the Ca-alginate matrix. Specifically, bands at 1420 cm^−1^ (symmetric stretching of COO^−^), as well as 1563 and 1615 cm^−1^ (asymmetric stretching of COO^−^), correspond to the carboxylate groups coordinated with Ca^2+^ in the egg-box structure [42]. These latter bands are associated with M-rich segments and G-block egg-box domains, respectively. The broad absorption band centered at approximately 3400–3500 cm^−1^ is attributed to O–H stretching vibrations associated with hydroxyl groups and adsorbed water. In the low-wavenumber region, bands between 487 and 770 cm^−1^ are assigned to phosphate bending vibrations (ν_2_ and ν_4_ of PO_4_^3−^), together with skeletal C–O–C/C–C deformations and Ca–O interactions [43]. The characteristic phosphate stretching vibrations (ν_1_ and ν_3_ of PO_4_^3−^), typically observed near 960 and 1030–1090 cm^−1^, are not clearly resolved because they overlap with the intense C–O stretching vibrations of the alginate matrix.

### 2.5. Scanning Electron Microscopy (SEM)

**Figure 4:**
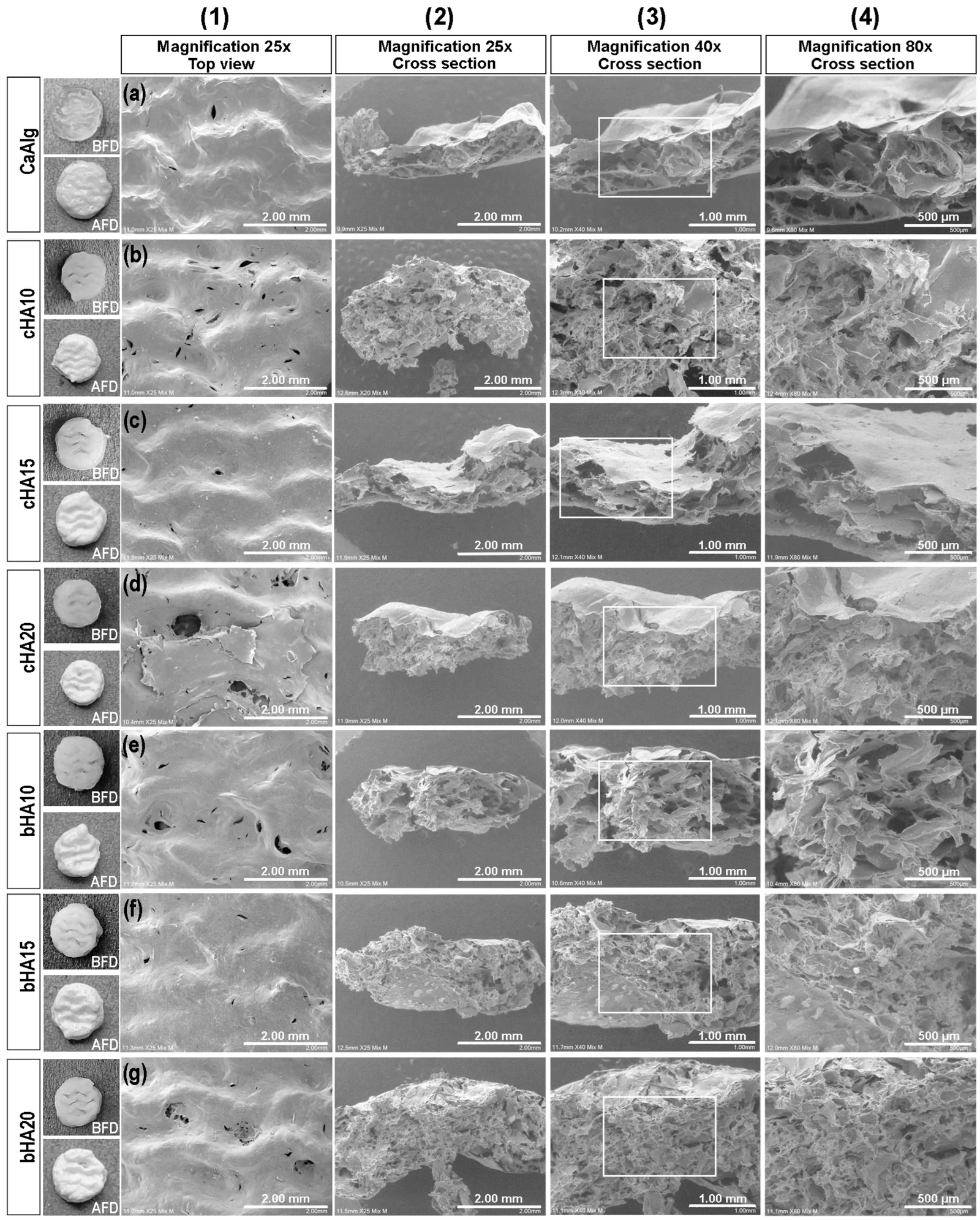
Representative photographs and SEM micrographs of the 3D-printed scaffolds. Insets in the first column show representative photographs of each scaffold before freeze-drying (BFD, Before Freeze-Drying) and after freeze-drying (AFD, After Freeze-Drying). SEM images depict the surface morphology (top view) and the internal architecture observed in cryo-fractured cross-sections. The white boxed area in the 40× cross-sectional images indicates the region shown at higher magnification (80×) in the corresponding images of the fourth column. Rows correspond to the experimental groups: (**a1**–**a4**) CaAlg, (**b1**–**b4**) cHA10, (**c1**–**c4**) cHA15, (**d1**–**d4**) cHA20, (**e1**–**e4**) bHA10, (**f1**–**f4**) bHA15, and (**g1**–**g4**) bHA20. Columns correspond to (1) top view (25×), (2) cross-sectional view (25×), (3) cross-sectional view (40×), and (4) cross-sectional view (80×).

The morphology of the scaffolds was evaluated by SEM after lyophilization (Figure 4). For these scaffolds, a gyroid-type infill was chosen because it offers superior mechanical properties, enhances cell adhesion, and increases mineralized matrix deposition compared to other infills [44]. From the top view, it can be observed that all inks showed a slight coalescence of their layers, but still maintained the topography of the gyroid infill. In the sample with the highest concentration of cHA, macropores were still formed, even though total coalescence of the printed layers did not occur. For samples obtained with bHA, increasing its concentration improves print resolution, making the gyroid infill clearer. The cross-sections after cryofracture were also analyzed, and macropores were observed in all samples. Increasing the hydroxyapatite concentration in both cases resulted in a denser structure, with reduced pore size and interconnectivity. A qualitative evaluation of the SEM micrographs indicated that the average pore size progressively decreased with increasing hydroxyapatite content. The CaAlg scaffold exhibited interconnected pores of approximately 120–220 µm. The cHA10, cHA15, and cHA20 scaffolds presented estimated pore sizes of approximately 80–180 µm, 60–140 µm, and 40–120 µm, respectively. Likewise, the bHA10 scaffold exhibited the largest interconnected pores, approximately 150–300 µm, whereas bHA15 and bHA20 presented pore sizes of approximately 70–180 µm and 40–100 µm, respectively. According to Picado-Tejero et al. [45], porous and interconnected structures can improve biological activity by facilitating the diffusion of nutrients throughout the structure. Therefore, the amount of hydroxyapatite can be a key factor in altering biological performance, as changes in scaffold porosity can affect it.

### 2.6. Mechanical Characterization

**Figure 5:**
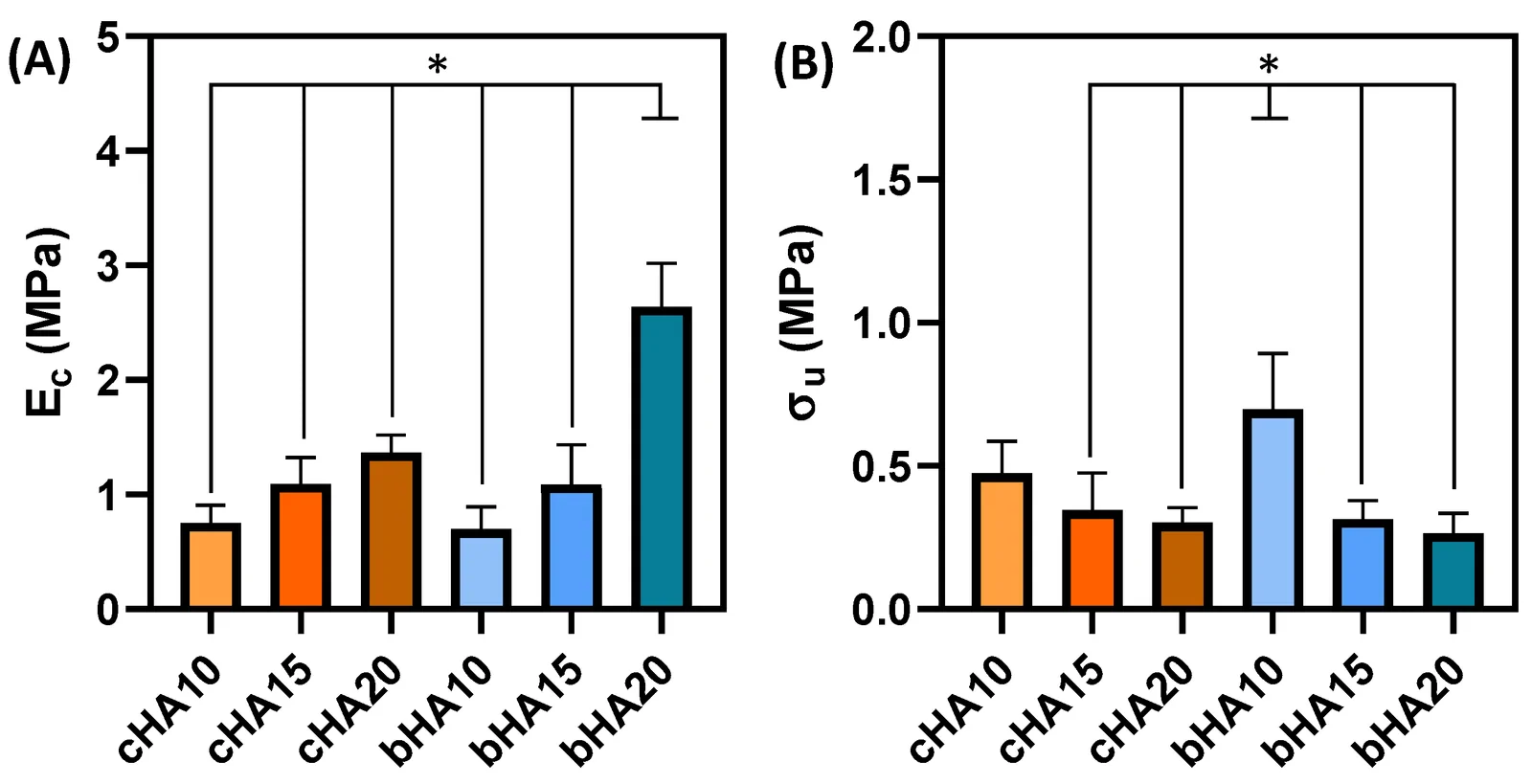
(**A**) Compressive modulus (E_c_) and (**B**) maximum compressive strength (σ_u_) results for cHA and bHA scaffolds. Results are presented as mean ± standard deviation (*n* = 9). Statistical significance was determined by one-way ANOVA with post hoc test, where * *p* < 0.05.

The mechanical properties of the scaffolds were evaluated through compression tests, including the compressive modulus (E_c_) and the maximum compressive strength (σ_u_) (Figure 5). E_c_ is related to the material’s stiffness during compression, and in bone tissue engineering, it is important for assessing the mechanical compatibility with the host bone and providing structural support during the repair process [46]. The E_c_ value tends to increase with increasing hydroxyapatite content in both cases. The E_c_ values for samples with 10% and 15% of both hydroxyapatites are similar (~0.7 MPa for 10% and ~1.1 MPa for 15%), but for samples with 20%, the values differ, with cHA20 having an E_c_ of 1.37 MPa and bHA20 having an E_c_ of 2.64 MPa. This difference arose from morphological differences between the two types of ceramics. Kane and Roeder observed differences in the mechanical properties of collagen scaffolds with different hydroxyapatite morphologies (whisker and powder), with whiskers exhibiting higher E_c_ values [47]. The σ_u_ value is related to the scaffold’s load-bearing capacity. It was observed that this value decreases in both cases as the hydroxyapatite concentration increases. For cHA, this value decreases from 0.47 to 0.30 MPa, while for bHA it decreases from 0.69 MPa to 0.26 MPa. This behavior is associated with an increase in the ceramic fraction and a reduction in structural porosity, making the scaffold susceptible to collapse under compression.

### 2.7. Degradation Behavior and Surface Mineralization in SBF

**Figure 6:**
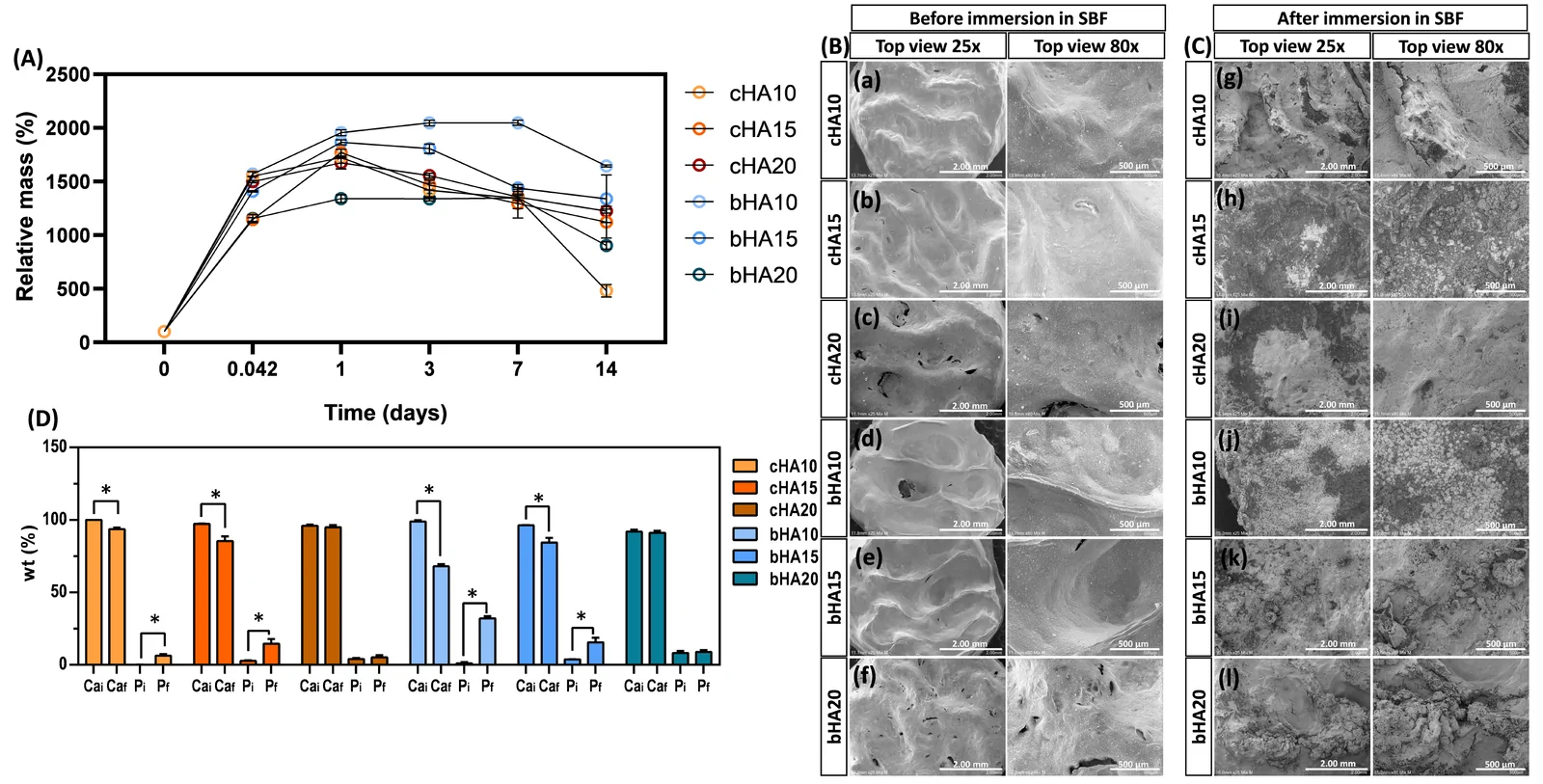
(**A**) Relative mass variation of the samples immersed in simulated body fluid (SBF). (**B**) Representative SEM micrographs of the surface morphology (top view) of scaffolds before immersion in SBF. Rows correspond to the experimental groups: (**a**) cHA10, (**b**) cHA15, (**c**) cHA20, (**d**) bHA10, (**e**) bHA15, and (**f**) bHA20. Within each row, column 1 corresponds to the 25× top-view image and column 2 corresponds to the 80× top-view image. (**C**) Representative SEM micrographs of the surface morphology (top view) of scaffolds after 14 days of immersion in SBF. Rows correspond to the experimental groups: (**g**) cHA10, (**h**) cHA15, (**i**) cHA20, (**j**) bHA10, (**k**) bHA15, and (**l**) bHA20. Within each row, column 1 corresponds to the 25× top-view image and column 2 corresponds to the 80× top-view image. (**D**) Semi-quantitative elemental composition obtained by XRF analysis before and after immersion in simulated body fluid (SBF). Ca_i_ and P_i_ represent the calcium and phosphorus contents before immersion, whereas Ca_f_ and P_f_ correspond to the calcium and phosphorus contents after immersion in SBF. The results are presented as mean ± standard deviation (*n* = 3). Statistical significance was determined by one-way ANOVA with post hoc test, where * *p* < 0.05.

The stability of the scaffolds after lyophilization was analyzed for up to 14 days in SBF at 37 °C (Figure 6A). For all samples, a peak in relative mass increase was observed on the first day, likely due to the high water absorption of these materials. For the cHA samples, the maximum absorption occurred after the first day, whereas for the bHA samples, it occurred between 3 and 7 days. After reaching their maximum, all samples showed a reduction in mass on the 14th day, likely due to scaffold degradation. The slow degradation of these scaffolds is very important because, initially, they provide structural support for the repair process and, over time, are gradually replaced by newly repaired tissue [48]. Furthermore, changes related to cHA or bHA are observed in the scaffolds. Scaffolds with cHA did not show differences in their stability over time, with only the cHA10 sample showing the greatest mass loss after the 14 days. For the samples containing bHA, hydration and degradation were observed to be inversely proportional to the bHA content, such that lower concentrations resulted in higher hydration. Specifically, bH10 reached relative mass increase values close to 2000%, whereas bHA15 and bHA20 reached values of approximately 1800% and 1300%, respectively. This behavior is due to the alteration in porosity observed by SEM analysis. The statistical differences indicate that the bHA10 sample indeed exhibits behavior significantly different from that of the other samples, as shown in the heatmap analysis (Figure S1).

The morphology of the scaffolds was also analyzed before and after SBF immersion (Figure 6B,C). Before immersion, a practically intact surface was observed, without artifacts. However, after 14 days, alterations resulting from degradation and interaction with SBF were observed. The surface of all samples showed a defective topography, presenting cracks and unevenness; in addition, the deposition of various surface artifacts was observed. In particular, a greater deposition of artifacts was noted for the cHA15 and bHA10, evidenced by the appearance of particles with greater spectroscopic contrast than the polymeric base. These artifacts may originate from the deposition of an apatite-type layer on the scaffold surface, a phenomenon typically observed in bioactive materials exposed to SBF. According to Dridi et al., apatite deposition occurs through a stepwise process in which amorphous calcium phosphate phases initially form and reorganize through ion exchange with the environment, evolving into apatite nuclei that subsequently grow and mature into a bone-like apatite layer [49]. Cao et al. observed similar results using Ti substrates coated with fluorine–hydroxyapatite and immersed in SBF [50].

To confirm the SEM results, XRF measurements were performed before and after immersion of the scaffolds in SBF (Figure 6D). Elemental analysis by XRF performed before and after 14 days of incubation in SBF revealed an overall increase in P content, accompanied by a slight reduction in calcium levels in the scaffolds. The samples containing 20% hydroxyapatite (cHA or bHA) did not show significant changes in Ca and *p* values, probably because of the denser structure observed by SEM. The greatest increase in P and subsequent decrease in Ca were observed for the bHA10 sample, due to the interconnected microporous structure and water absorption capacity of this sample. This behavior confirms the occurrence of ion exchange processes between the material and the medium, in which PO_4_^2−^ ions from the SBF are incorporated into the scaffold matrix, while some of the originally present Ca^2+^ ions are released or structurally reorganized, forming the initial stages of apatite-like phase deposition on the scaffold surface. When the ability to alter the local pH was evaluated, all samples slightly reduced the pH at the end of 14 days, reaching levels between 6.7 and 7 (Figure S2). This occurs because calcium alginate degrades, releasing some of its Ca^2+^ ions from its egg-box structure into the medium [51].

### 2.8. Metabolic Activity and Cell Proliferation

The selection of the pre-osteoblast MC3T3-E1 cell line was based on its widespread use in studies on scaffold development for bone regeneration and repair, due to its well-recognized ability to produce a mineralized extracellular matrix [52,53,54]. Moreover, this cell line is widely accepted as an appropriate model for evaluating biocompatibility and osteogenesis, as it reproduces fundamental cellular behaviors involved in bone regeneration, including adhesion, proliferation, viability, and osteoblastic differentiation.

**Figure 7:**
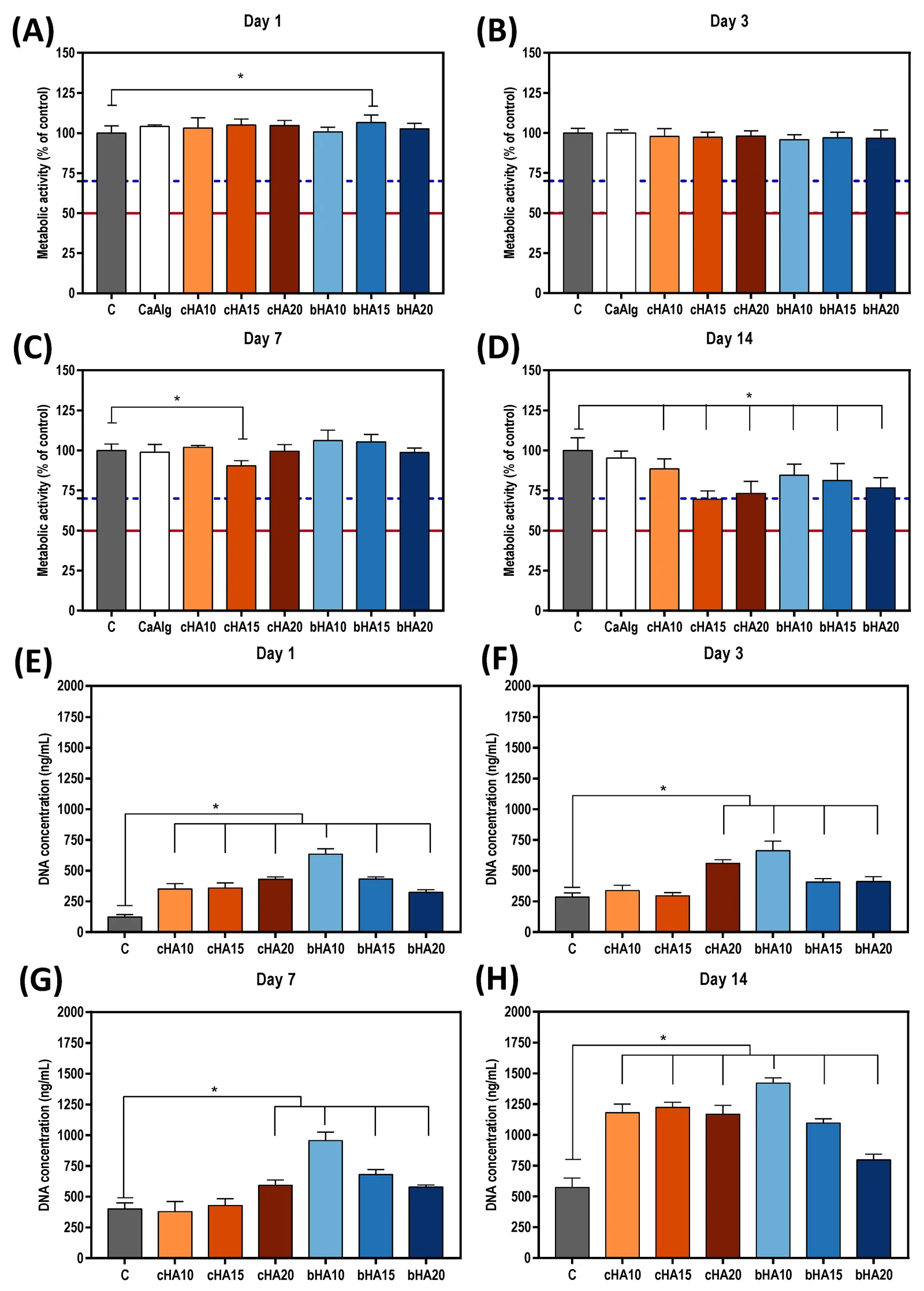
(**A**–**D**) Metabolic activity assessed by the Alamar Blue assay on days 1, 3, 7, and 14, respectively. Data were normalized to the control. The blue horizontal line indicates the 70% cell viability threshold established by ISO 10993-5:2009 for classifying materials as non-cytotoxic [56]. The red horizontal line (50% cell viability) is included as a visual reference. Accordingly, viability values below 50% indicate severe cytotoxicity, whereas values between 50% and 70% indicate moderate cytotoxicity. (**E**–**H**) Quantification of total DNA content (ng/mL) on days 1, 3, 7, and 14 after exposure to the conditioned media, respectively. DNA concentration was determined based on a standard calibration curve. Results are presented as mean ± standard deviation (*n* = 6). Statistical analysis was performed using one-way ANOVA followed by a post hoc test; differences were considered statistically significant at * *p* < 0.05.

The metabolic activity of MC3T3-E1 cells was evaluated using the Alamar Blue assay as an indirect indicator of biocompatibility, since only viable cells exhibit sufficient metabolic activity to reduce resazurin, a blue, non-fluorescent oxidized molecule, into resorufin, its highly fluorescent pink reduced form [55]. This reaction is catalyzed by oxidoreductase enzymes associated with cellular energy metabolism, predominantly localized in the mitochondrial respiratory chain, although they are also present in the cytoplasm and the endoplasmic reticulum. Thus, this assay reflects the overall cellular redox state and general metabolic condition, as illustrated in Figure 7A–D.

As shown in Figure 7A,B, after 1 and 3 days of exposure, all composites with cHA or bHA showed values very close to 100% compared with the negative control, with no significant differences between the groups. This underscored the fact that they were cytocompatible, and no negative influence was exerted by any species released on cellular metabolism. In Figure 7C, on day 7, metabolic activity was significantly reduced only in the cHA15 sample compared with the control. All other groups had values above 99%. At this time point, cells treated with bHA at 10% and 15% showed slightly increased metabolic activity (106% and 105%, respectively). However, such increases were not statistically significant.

On the other hand, in Figure 7D, a decrease in metabolic activity is observed in all experimental groups, except the CaAlg sample, after 14 days of exposure. Greater reductions occurred in the scaffolds with cHA15 and cHA20, yielding metabolic activity values of 70% and 73%, respectively, whereas the cHA10 group showed a higher value of 89%. A less pronounced reduction was observed with bHA-containing scaffolds, with metabolic activity values of 84%, 81%, and 76% at 10%, 15%, and 20%, respectively. In turn, an inverse relationship between the content of bHA and the reduction in metabolic activity after a long exposure period was observed. Such behavior can be explained by variation in the release of water-soluble components between these tested scaffolds, resulting from their internal architecture, as seen by SEM.

Despite the overall trend toward reduced metabolic activity observed for both hydroxyapatites, all experimental groups maintained viability values above 70%*,* the threshold defined by ISO 10993-5:2009 for non-cytotoxic materials [56]. These findings are consistent with and reinforce previously reported data by Prado et al. [57], who demonstrated enhanced metabolic activity in MC3T3-E1 cells exposed to hydroxyapatite derived from *Micropogonias furnieri* compared with a control group containing only conventional culture medium, with statistically significant differences observed after 3 and 6 days of exposure. Shi et al. also reported superior biological compatibility of hydroxyapatite extracted from trout and salmon bones when compared to synthetic hydroxyapatite in MC3T3-E1 osteoblast cultures [24].

The micrographs obtained from the indirect metabolic activity assay (Figure S3) further corroborated these findings, as no morphological alterations indicative of cellular stress or cell death were observed. The cells exhibited an elongated appearance typical of their type, with clear intercellular connections and layered growth. This verification of cell morphology is very important, since substantial evidence in the literature indicates a direct link between cell shape characteristics and the physiological metabolic state [58,59,60,61].

Metabolic activity should not be considered an isolated or direct indicator of cell proliferation. An increase in metabolic activity primarily reflects greater efficiency in intracellular energy production and cell viability at a given time point; proliferation can only be reliably inferred through complementary assays, such as DNA quantification or direct cell counting. Therefore, for a more comprehensive assessment of the effects of the scaffolds on cell viability and proliferation, these two parameters were evaluated using complementary approaches: the Alamar Blue assay and fluorescence-based DNA quantification with PicoGreen^®^ [62]. After 1 day of exposure (Figure 7E), a statistically significant increase in DNA content was observed in almost all groups compared to the control, with values close to threefold. The group containing bHA10 showed the most pronounced increase, reaching approximately five times the DNA content of the control. By this time, an inverse relationship between bHA content and DNA levels could be observed with values of 633.82, 433.09, and 323.64 ng/mL for the samples containing bHA10, bHA15, and bHA20, respectively. For treatments containing cHA10, cHA15, and cHA20, DNA values were 350.55, 358.93, and 429.77 ng/mL, respectively, indicating a similar proliferative response across these formulations.

After 3 days of exposure (Figure 7F), conditioned medium derived from scaffolds containing 10% and 15% cHA did not significantly affect DNA content compared with the control group (285.97 ng/mL). In contrast, conditioned medium from 20% cHA scaffolds significantly enhanced cell proliferation, increasing DNA levels to 559.79 ng/mL, approximately twofold higher than the control. Among the bHA-containing scaffolds, an inverse relationship between ceramic content and DNA levels was observed, with all groups showing significantly higher values than the control. The greatest proliferative response was detected in the 10% bHA group (662.36 ng/mL), followed by the 15% and 20% formulations. After 7 days of exposure (Figure 7G), this response became more pronounced. Within the cHA groups, only the 20% formulation maintained a significantly higher DNA content (593.29 ng/mL) than the control (400.48 ng/mL). In contrast, the inverse relationship among the bHA groups remained evident, with DNA contents of 956.21, 679.54, and 577.85 ng/mL for scaffolds containing 10%, 15%, and 20% bHA, respectively. By day 14 (Figure 7H), all conditioned media significantly increased DNA content relative to the control, with values approaching a twofold increase. The 10% bHA group again exhibited the highest proliferative response, reaching 1421.25 ng/mL, approximately 2.5-fold greater than the control. The inverse relationship between bHA concentration and DNA content persisted, with values progressively decreasing to 1096.24 and 796.68 ng/mL for the 15% and 20% bHA groups, respectively. In contrast, conditioned media derived from 10%, 15%, and 20% cHA scaffolds produced comparable proliferative responses, with DNA contents of 1180.38, 1222.90, and 1168.10 ng/mL, respectively.

The more pronounced proliferative response observed in the bHA10 sample than in the cHA samples may be related to its higher porosity and water absorption capacity. These features likely favored both the leaching of water-soluble components and the diffusion of nutrients into the scaffold. This interpretation is supported by the scaffold stability and SEM results. This proliferative effect observed for scaffolds containing bHA is consistent with the previous work of Mondal et al. [63], who compared the proliferative effects of hydroxyapatite extracted from fish bones (tuna) with those of synthetic hydroxyapatite and reported superior cell proliferation on biogenic hydroxyapatite. Although Mondal et al. attributed this behavior to the presence of trace elements, no elemental analysis beyond Ca and P was performed in the present study. Therefore, no such relationship can be established here. Complementarily, the present results are consistent with the study by Fang et al. [64], who reported higher proliferative activity in cells exposed to *Carassius auratus* scales compared with a negative control. In that study, the control group exhibited approximately 37% proliferative activity, whereas the experimental group reached approximately 60.3%.

### 2.9. Evaluation of Intracellular Nitrosative and Oxidative Stress

Oxidative stress is involved in several pathological conditions; however, it also plays fundamental roles in the regulation of physiological cellular activities [65]. In this context, the production of reactive species has been widely connected with the interaction between tissues and implanted biomaterials, being intrinsically linked to the host’s response to these materials. Both reactive oxygen species (ROS) and reactive nitrogen species (RNS), as well as lipid peroxidation products, act as chemoattractants and signaling molecules in the recruitment and activation of inflammatory cells during wound healing. These species also act as modulatory and degradative agents during the inflammatory and reparative phases, contributing to the remodeling and maturation of the extracellular matrix at the wound site. This factor can directly impact the healing process, underscoring the need to monitor reactive species release given their dual, concentration-dependent biological effects.

**Figure 8:**
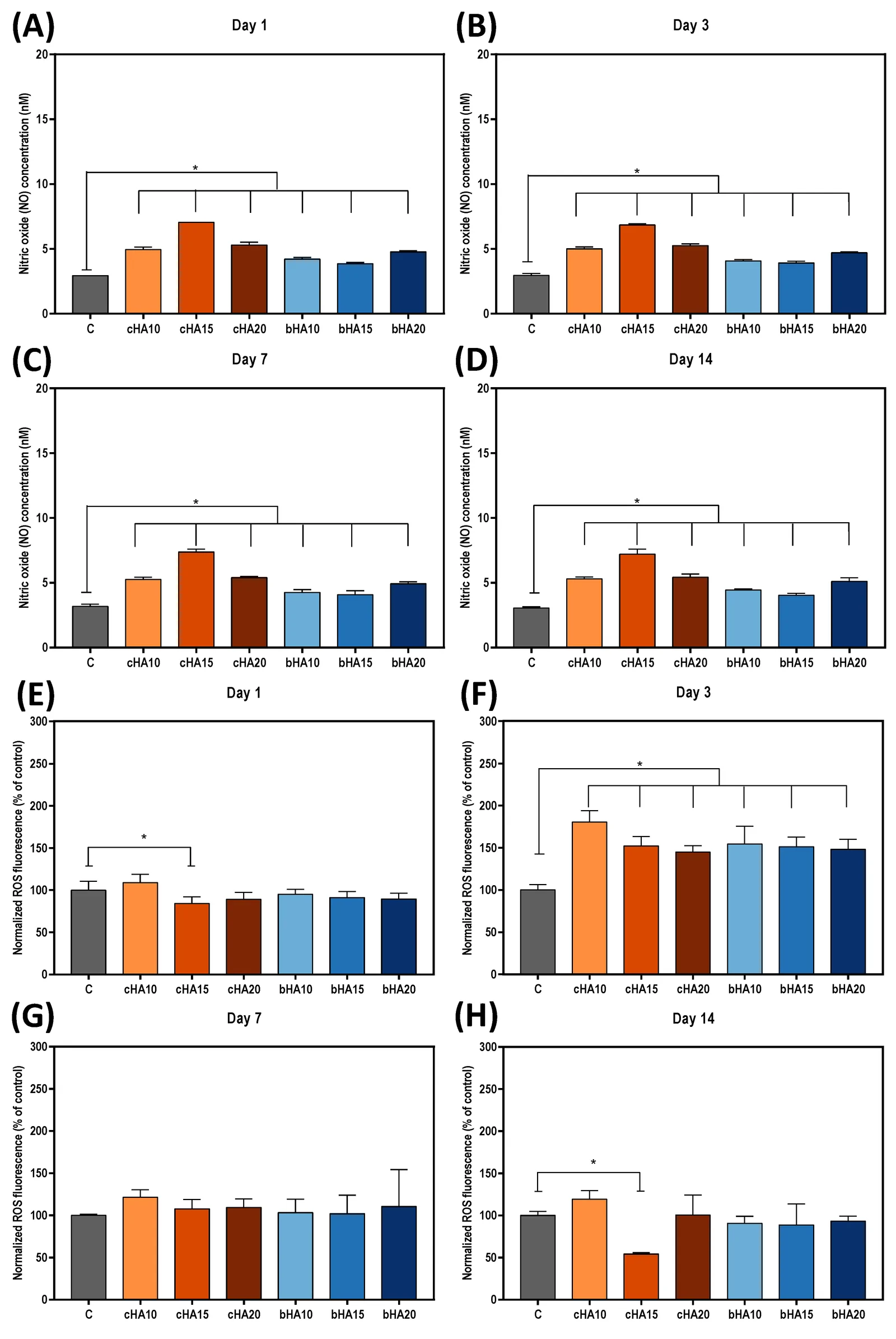
(**A**–**D**) Extracellular RNS levels, expressed in nM, evaluated at 1, 3, 7, and 14 days after exposure to the conditioned media. NO quantification was performed based on a standard calibration curve. (**E**–**H**) Intracellular ROS levels determined by the DCFDA assay after 1, 3, 7, and 14 days of exposure to the conditioned media, respectively. Relative fluorescence values were normalized to the control group. Results are presented as mean ± standard deviation (*n* = 6). Statistical analysis was performed using one-way ANOVA followed by a post hoc test. Statistically significant differences were considered at * *p* < 0.05.

Results in Figure 8A–D show a statistically significant increase in NO production compared to the control group. Generally, NO levels were maintained near the basal levels of the control group, indicating that at these concentrations, NO may act as a stimulator of cell survival, growth, and differentiation [66]. No exacerbated increase was found at any time point during the experiment; values did not exceed 10 nM. There was no dose-dependent pattern in any of the groups, whether cHA or bHA was present or not, but a slightly higher increase of about 7 nM was noted in the group that was exposed to conditioned medium containing cHA15 scaffolds. Lee et al. reported that NO levels within the range of 1–30 nM are considered biologically active, capable of inducing angiogenesis, cell survival, and proliferation via activation of the soluble guanylate cyclase (sGC) pathway [67]. The results obtained here show good correlation with the biocompatible profiles observed for the different groups at 1-, 3-, 7-, and 14-day exposure periods.

ROS include free radicals and non-radical species that are byproducts of cellular metabolism [65,68]. Similar to RNS, ROS exhibit a dual concentration-dependent role: at low levels, they act as signaling mediators; at moderate levels, they are physiologically relevant during the inflammatory and proliferative repair phases; and, when in excess, they induce oxidative stress, leading to cellular damage [69]. Analysis of intracellular ROS levels (Figure 8E–H) indicated that, on the first day after exposure to conditioned media containing cHA and bHA (Figure 8E), ROS levels did not vary much from their baseline state, represented by the control group. On the third day after exposure (Figure 8F), a transient increase was observed. This increase can be considered an initial adaptive response [70]. At later exposure times of 7 and 14 days (Figure 8G–H), ROS levels returned to near-normal levels, showing that homeostasis was restored over time. The only exception was the cHA15 group after 14 days of exposure (Figure 8H), which showed lower ROS levels than the control. Overall, this behavior is not compatible with a scenario of persistent or cytotoxic oxidative stress.

### 2.10. Cell Adhesion and Migration

**Figure 9:**
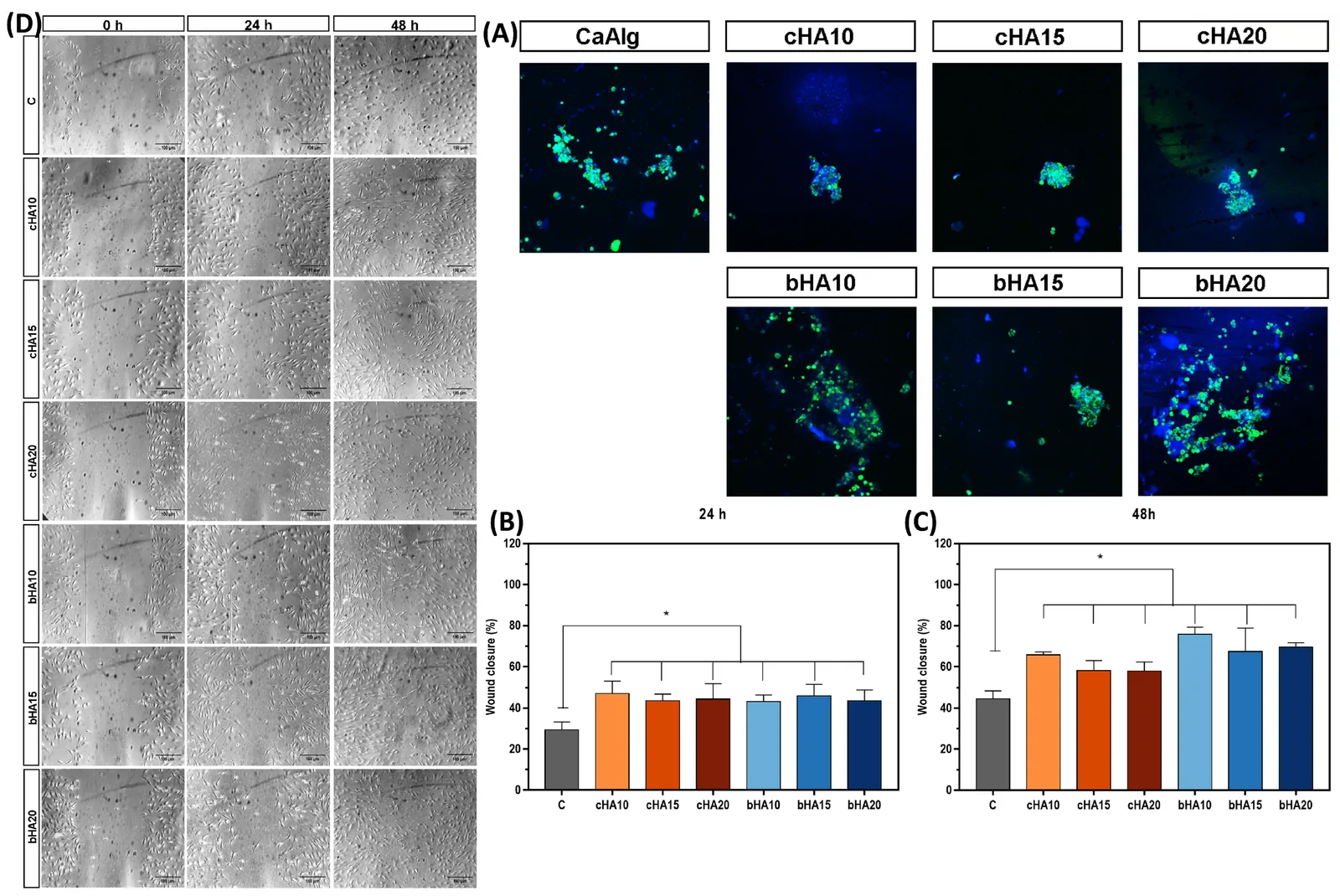
(**A**) Confocal optical microscopy images of adhered cells after 1 day of contact, highlighting nuclear staining with DAPI (blue) and cytoskeletal organization visualized by phalloidin (green). (**B**,**C**) Quantification of wound closure corresponding to the images obtained in the cell migration assay. Results are presented as mean ± standard deviation (*n* = 3). Statistical significance was determined by one-way analysis of variance (ANOVA), followed by a post hoc test; differences were considered statistically significant when * *p* < 0.05. (**D**) Representative images of the wound healing assay at different experimental time points (24 h and 48 h).

Cell adhesion of MC3T3-E1 cells on the surface of scaffolds fabricated with bHA and cHA was evaluated by confocal fluorescence microscopy after 1 day of culture (Figure 9A). For visualization of the actin cytoskeleton and cell nuclei, Alexa Fluor 488-conjugated phalloidin and DAPI were used, respectively, enabling the assessment of cell morphology and cell–material interactions on the scaffold surfaces. The micrographs obtained for all experimental groups clearly demonstrate cell adhesion on the scaffold surfaces, indicating that none of the components present in the formulations adversely affected this initial process. However, a qualitatively higher apparent number of adhered cells was observed on scaffolds containing bHA compared with those containing cHA. These findings are consistent with the study by Pon-On et al. [71], who reported enhanced adhesion of UMR-106 osteoblastic cells on surfaces containing hydroxyapatite extracted from *Puntius jullieni* fish scales compared with synthetic hydroxyapatite. A similar trend was described by Baek et al. [72], who observed increased cell adhesion on scaffolds composed of polycaprolactone (PCL) and hydroxyapatite derived from marine plankton exoskeletons. The authors attributed this behavior primarily to increased surface roughness and favorable interactions between marine-derived hydroxyapatite surfaces and bone cells.

The migratory profile of MC3T3-E1 osteoblasts was assessed indirectly by the scratch wound assay, using cells in the presence of conditioned media obtained from the incubation of scaffolds with cHA and bHA. Untreated control cells showed approximately 29% and 44% closure after 24 and 48 h, respectively, establishing a baseline migratory profile under standard cell culture conditions, and were therefore used as a reference (Figure 9B–D). A statistically significant increase compared with the control was observed for all treated groups at both time points for cHA- and bHA-conditioned media. After 24 h of exposure (Figure 9B), the cHA10, cHA15, and cHA20 groups exhibited wound closure percentages of approximately 47%, 44%, and 44%, respectively, whereas the bHA10, bHA15, and bHA20 groups showed values of 43%, 46%, and 44%. After 48 h (Figure 9C), this enhancement in the migratory profile became more pronounced compared to the control, with wound closure percentages of 66%, 58%, and 58% for cHA10, cHA15, and cHA20, respectively. Similarly, cells exposed to bHA-conditioned media exhibited even higher wound closure percentages, corresponding to 76%, 67%, and 69% for bHA10, bHA15, and bHA20, respectively.

These findings are consistent with the study by Fan et al. [73], in which the authors reported an enhanced migratory profile of HUVECs after 6 and 12 h of exposure to different scaffolds containing Ce-doped hydroxyapatite nanowires. In that study, the group exposed to the GDM/CeHA@CA scaffold exhibited the most pronounced increase in cell migration rates, reaching 13.11% wound closure at 6 h and 30.03% at 12 h, an effect attributed to the synergistic action of Ce^3+^/Ce^4+^, deferoxamine, and Mn^2+^. In the same work, the migratory capacity of hBMSCs was also evaluated, and exposure to the GDM/CeHA@CA scaffold resulted in a migration rate of 61.27% after 24 h. Similar results were reported by Sistani et al. [74], who developed PLA-based scaffolds containing nano-hydroxyapatite and demonstrated that groups containing 5% nHA exhibited a significant increase in wound closure after 24 h compared to the control, in addition to a substantial reduction in the cell-free gap area in the 10% nHA group.

### 2.11. Extracellular Matrix Mineralization

**Figure 10:**
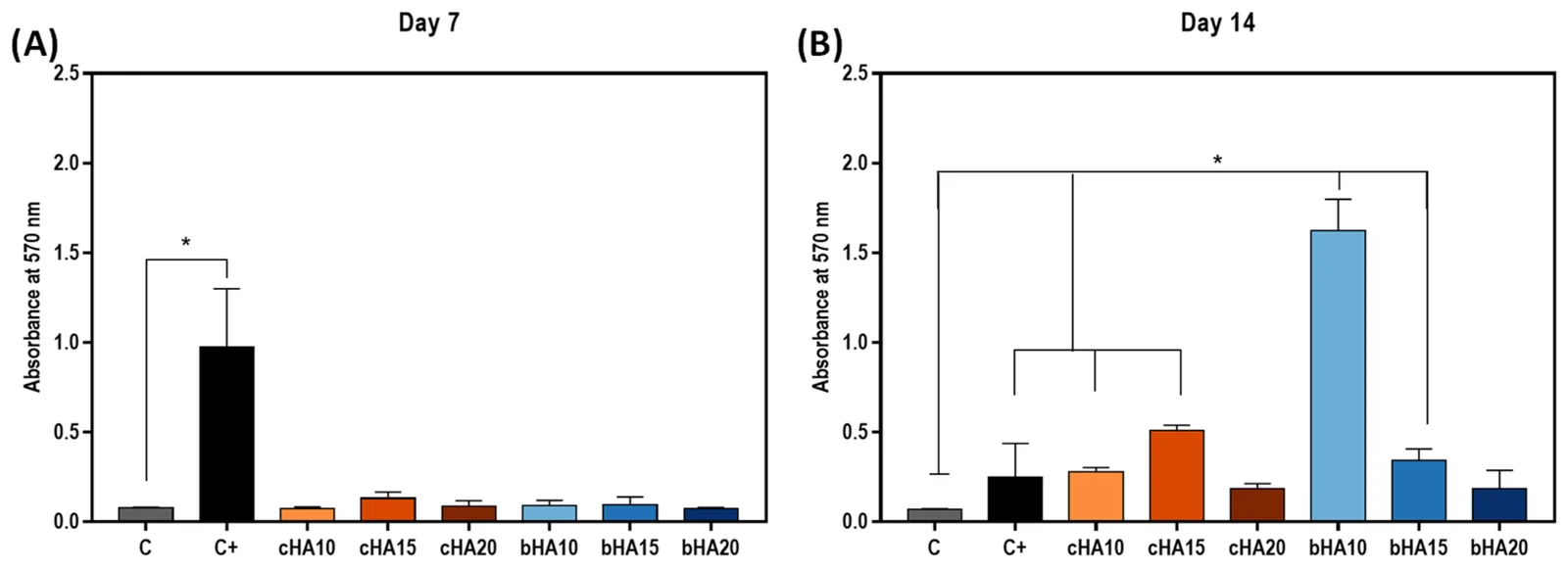
Quantitative analysis of mineralization assessed by ARS staining after (**A**) 7 and (**B**) 14 days of exposure to conditioned media containing cHA and bHA, respectively. Results are expressed as mean ± standard deviation (*n* = 6). Statistical analysis was performed using one-way ANOVA followed by a post hoc test, differences were considered statistically significant when * *p* < 0.05.

The assessment of extracellular matrix mineralization using Alizarin Red S (ARS) staining is one of the most widely employed standards for detecting calcium deposits associated with osteoblast osteogenic differentiation in vitro, reflecting the cells’ ability to form a mineralized matrix resembling mature bone [75]. In this context, the results obtained after 7 days of exposure indicated that only the control group supplemented with osteogenic medium exhibited a detectable signal of extracellular mineralization (Figure 10A), suggesting that osteoblastic differentiation had not yet been sufficiently stimulated by the soluble biomolecules released from the conditioned media of cHA- or bHA-containing scaffolds at this early stage.

But after 14 days of exposure, as revealed in Figure 10B, a statistically significant increase in extracellular matrix mineralization was observed in the positive control group and in cells exposed to treatments with cHA10-, cHA15-, bHA10-, and bHA15-conditioned media, compared to the negative control group without osteogenic stimulation. With increasing cHA content in the scaffolds, mineralization showed a directly proportional trend, with higher percentages of this ceramic material presumably favoring greater deposition of the mineralized matrix. However, as bHA content increased in the scaffolds, the mineralization signal showed an inverse relationship, with bHA10 exhibiting the highest response, which gradually decreased with increasing bHA content. This trend correlates with the XRF results obtained after immersing the respective scaffolds in SBF for 14 days, as the semiquantitative analysis revealed variations in the relative Ca and P contents. It is worth noting that the P content increased, possibly linked to surface enrichment due to the deposition of apatite-like mineral phases. This was more evident in the case of scaffolds containing bHA10. Other similar profiles for P enrichment were also found in scaffolds containing cHA10, cHA15, bHA10, and bHA15. These results indicate the occurrence of ionic exchange interactions between the scaffolds and the SBF, which may contribute to the formation of a superficial apatite layer. Although apatite formation in SBF represents a predominantly physicochemical phenomenon, distinct from osteoblast-mediated biological mineralization, several studies indicate that a material’s ability to form apatite in SBF can be used as an indicator of its potential to provide a surface conducive to mineral deposition under physiological conditions [76].

In this context, the results of the present study are consistent with those reported by Venkatesan et al. [25], who demonstrated that nano-hydroxyapatite isolated from salmon bones promoted a higher degree of mineralization (118%) compared with both the group exposed only to osteogenic medium (104%) and the control group (100%), indicating the superior performance of this biogenic hydroxyapatite source in inducing mineral deposition by mesenchymal stem cells. A similar behavior was reported by Yousefi et al. [77], who observed increased mineralization in MC3T3-E1 cell cultures treated with nano-hydroxyapatite extracted from carp bones and human bones compared with cells treated with commercial nano-hydroxyapatite.

### 2.12. Genotoxicity

Although hydroxyapatite is widely used in the development of biomedical devices and implants, studies evaluating the potential for genotoxic events associated with this ceramic and its derived materials are still scarce. Investigating these materials is essential to ensure their safety before their application in more complex biological systems, such as in vivo studies [78]. The genotoxic potential of scaffolds containing cHA and bHA was evaluated using the micronucleus assay, a widely used method for detecting DNA damage and chromosomal instability. This approach is fundamental to ensuring the safety of biomaterials used in the development of implants for the treatment of critical fractures, as it allows the identification of whether components eventually released from the scaffolds are capable of inducing DNA damage or causing genomic instability in surrounding tissues.

**Figure 11:**
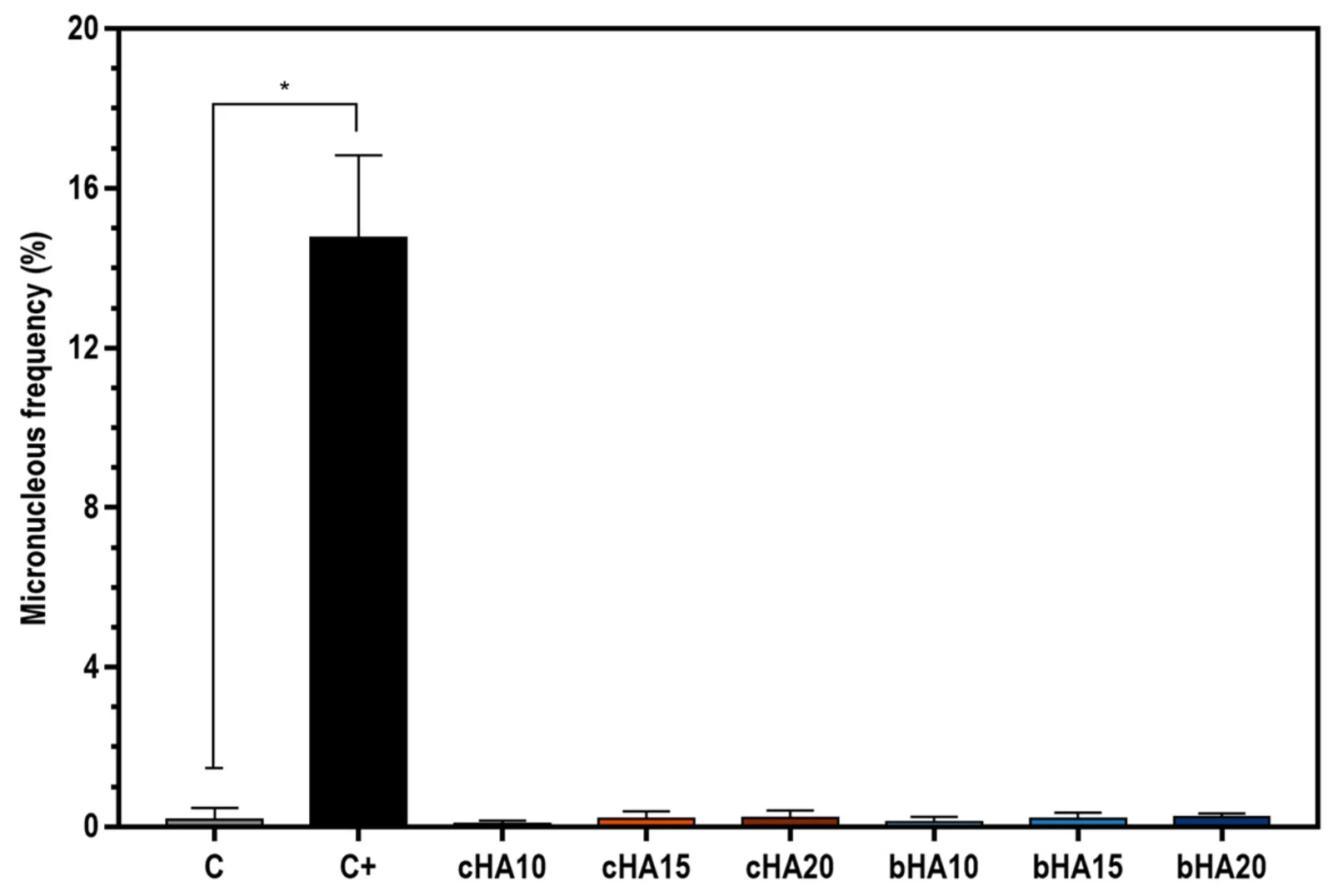
Micronucleus assay for the CHO-K1 cell line after 24 h of exposure to conditioned media with cHA and bHA. The results are presented as mean ± standard deviation (*n* = 6). Statistical significance was assessed using one-way ANOVA with post hoc tests, with * *p* < 0.05 indicating statistical significance.

According to the results presented in Figure 11, none of the groups exposed to conditioned media containing cHA or bHA showed micronucleus frequencies higher than 0.23%, values lower than those observed in the negative control (1.73%). In contrast, a statistically significant difference was observed only for the positive control, treated with camptothecin (1 µM), which exhibited a high micronucleus frequency (14.77%), confirming the assay’s sensitivity. These findings are consistent with the results reported by Yamamura et al., who observed no genotoxic effects after 30 days of exposure in liver, kidney, and lung tissues, as well as in blood cells of Wistar rats [79]. In that study, HA fragments, with an area of 0.5 cm^2^, extracted from *Micropogonias furnieri*, were implanted in the animals, and genotoxicity was assessed using the comet assay. The authors reported no genotoxicity following implantation of the material. Similar findings were also reported by Kido et al. [80], who evaluated the genotoxic potential of alumina scaffolds coated with cHA, identifying no genotoxicity in liver and kidney tissues or in blood cells. These results corroborate the safety of using both types of HA evaluated in this study, under comparable experimental conditions. Taken together, the results of this study reinforce those scaffolds developed with bHA10 do not exhibit genotoxic potential, are biocompatible, and are promising for promoting activities related to bone regeneration, highlighting their safety in biomedical and regenerative applications.

## 3. Materials and Methods

### 3.1. Extraction of Biogenic Hydroxyapatite from Fish Bones

Following the protocol proposed by Yamamura et al. [42], biogenic hydroxyapatite was obtained from the vertebral columns of *Micropogonias furnieri*, commercially acquired from a seafood market in the city of Santos, São Paulo, Brazil, under SISGEN authorization A034CEC. The species was selected for its suitable bone size, year-round availability, wide distribution along the Brazilian coastline, and its high commercial relevance [81]. Initially, muscle tissues were mechanically removed, and the bones were washed with distilled water and boiled for 1 h to facilitate the removal of residual tissues. Subsequently, the bones were subjected to alkaline treatment with 1 M sodium hydroxide (NaOH, (Synth, Diadema, Brazil, P.A.)) for 24 h, to denature and solubilize organic components, such as collagenous proteins, lipids, and fatty acids. After treatment, the alkaline solution was discarded, and the bones were washed with distilled water for 5 h to neutralize residual alkalinity. This procedure was repeated once, followed by at least three additional washing steps, ensuring complete material neutralization.

Thereafter, the bones were subjected to a bleaching step using hydrogen peroxide (H_2_O_2_, Synth, 30%) for 24 h, followed by three further washes with distilled water. Finally, the samples were calcined in a muffle furnace (EDG Equipamentos e Controles Ltda., São Carlos, Brazil) at 800 °C, with a heating rate of 5 °C/min and an isothermal dwell time of 5 h, yielding biogenic hydroxyapatite (bHA). To reduce variability in particle size between the biogenic hydroxyapatite and the commercial hydroxyapatite (cHA; Sigma-Aldrich, St. Louis, MO, USA; purity >90%) used as a reference material, both samples were subjected to wet planetary ball milling (MA 500, Marconi Equipamentos para Laboratório Ltda., Piracicaba, Brazil) for 15 min at 300 rpm until an average particle diameter of 10 µm was obtained.

### 3.2. Scaffold Fabrication

In this study, 3D scaffolds were fabricated by 3D printing. The inks were prepared from a 4% (*w*/*v*) sodium alginate solution in Milli-Q^®^ water (NaAlg, Sigma-Aldrich, #W201502), used as the polymeric matrix. To this matrix, bHA or cHA was added, with the latter being employed as a ceramic reference material widely used in the development of bone implants. The evaluated HA:NaAlg ratios were 10:100, 15:100, and 20:100 (*w*/*w*). Glycerol (0.25% *v/v*, Synth, Diadema, Brazil) was incorporated as a plasticizer, at a concentration selected to ensure biocompatibility and prevent interference with the scaffolds’ structural integrity. Homogenization was performed using an ultrasonic sonicator (QR 500, Eco-Sonics/Ultronique, Indaiatuba, Brazil) at 85% amplitude for 5 min until complete dispersion was achieved. Afterward, 12.2% (*v*/*v*) of a 2% (*m*/*v*) calcium chloride (CaCl_2_, 99.0%, Synth, Diadema, Brazil) solution was added to promote primary ionic crosslinking. The prepared inks were stored at 4 °C until printing.

Seven groups of experiments, as described in Table 1, were printed using an extrusion-based Octopus^TM^ 3D bioprinter (3D Biotechnology Solutions, Campinas, Brazil). The cylindrical geometry was modeled in Tinkercad^®^ with a diameter of 10 mm and a height of 4.5 mm, exported as an STL file, and later processed in PrusaSlicer^®^ using the parameters defined in Table 2. The linear extrusion print speed was set at 100 mm/min (1.67 mm/s) with an extrusion flow factor of 50%, defined in Pronterface, using a Straight-tip metal nozzle (not beveled) with a diameter of 1 mm.

Each scaffold exhibited a layer height of 0.6 mm, with seven layers required to reach the total height (Figure S4). After printing, a secondary ionic crosslinking step was performed by immersing the scaffolds in a 2% (*w*/*v*) calcium chloride solution for 10 min. Subsequently, the samples were washed with Milli-Q^®^ water, frozen overnight, and lyophilized for 72 h (−99 °C, 8.1 × 10^−4^ mbar), resulting in scaffolds with final dimensions of 5 mm in diameter and 1 mm in height. Before the in vitro assays, the scaffolds were freeze-dried (k105, Fisyka Biotecnologia, São Paulo, Brazil) for 72 h at −100 °C and sterilized by exposure to UV light for 4 h on both sides.

### 3.3. Physicochemical Characterization of Scaffolds

#### 3.3.1. X-Ray Diffraction (XRD)

The crystalline structures of the bHA and cHA powders, as well as the scaffolds fabricated from these materials, were determined by X-ray diffraction (XRD) using a DMax2500PC diffractometer (Rigaku, Japan) operated at 40 kV and 60 mA with Cu-Kα radiation (λ = 1.5406 Å). Data were collected over a 2θ range of 10–70°, using a scan rate of 1° min^−1^ and a step size of 0.01°. The obtained diffractograms were compared with reference patterns from the Powder Diffraction File (PDF) database.

The crystallinity index (CI) of the hydroxyapatite powders was estimated from the X-ray diffraction (XRD) patterns according to the method proposed by Landi et al. [34]. The CI was calculated using the intensity of the (300) diffraction peak (I_300_) and the minimum intensity of the valley between the (112) and (300) reflections (V_112/300_), according to Equation (1):

$$CI(\%)=\left(1-\frac{V_{112/300}}{I_{300}}\right)\times 100$$

where I_300_ corresponds to the maximum intensity of the (300) reflection and V_112/300_ is the minimum intensity measured between the (112) and (300) reflections. Higher CI values indicate a higher degree of crystalline order.

#### 3.3.2. Scanning Electron Microscopy (SEM) and Energy-Dispersive X-Ray Spectroscopy (EDS)

The surface morphology, topography, and elemental composition of the bHA and cHA powders, as well as the lyophilized scaffolds fabricated from these materials, were evaluated by scanning electron microscopy (SEM) coupled with energy-dispersive X-ray spectroscopy (EDS). Analyses were performed using a TM4000Plus II benchtop microscope (Hitachi High-Tech Corporation, Tokyo, Japan) equipped with an EDS detector and a LEO 1550 microscope (Carl Zeiss Microscopy GmbH, Oberkochen, Germany), operated at an accelerating voltage of 15 kV.

#### 3.3.3. Rheological Characterization

The rheological properties of the inks were evaluated using a modular compact rheometer (MCR-92, Anton Paar GmbH, Graz, Austria). All measurements were performed at 25 °C using a 25 mm parallel-plate geometry (PP25) with a fixed gap of 1 mm. The flow behavior was first investigated by obtaining flow curves using an up–down–up shear rate program ranging from 0 to 300 s^−1^ to evaluate the relationship between shear stress and shear rate. Oscillatory strain sweep tests were subsequently carried out from 0.01 to 10% strain at a constant frequency of 1 Hz to determine the linear viscoelastic region (LVR). Frequency sweep tests were then performed within the LVR using a fixed strain of 0.1% over a frequency range of 0.1–100 Hz to determine the storage modulus (G′) and loss modulus (G″). All measurements were performed in duplicate. Data acquisition was carried out using RheoCompass^TM^ software, while data processing and analysis were performed using Origin 2018 software.

#### 3.3.4. Fourier Transform Infrared Spectroscopy (FTIR)

The vibrational bands of the scaffolds were analyzed by Fourier transform infrared spectroscopy (FTIR) using an IRPrestige-21 spectrometer (Shimadzu Corporation, Kyoto, Japan) equipped with an attenuated total reflectance (ATR) accessory. Spectra were collected over the range of 600–4000 cm^−1^.

### 3.4. Mechanical Characterization

The compressive mechanical properties of the scaffolds (*n* = 9) were evaluated using an Instron 4444 universal testing machine (Instron, Norwood, MA, USA) equipped with a 1 kN load cell. A preload of 5 N was applied to ensure proper contact between the scaffold and the compression platens. Samples were compressed at a constant crosshead speed at room temperature until failure. Compressive stress–strain curves were recorded, and the compressive modulus (E_u_) and maximum compressive strength (σ_u_) were determined from the linear elastic region and the peak stress, respectively.

### 3.5. Relative Mass Change and pH Evaluation

The relative mass change in simulated Body Fluid (SBF, composition presented in Table S1) was determined at incubation periods of 1h (0.042 day), 1, 3, 7, and 14 days to check the stability of the lyophilized scaffolds (*n* = 6). Dry samples were weighed to get their initial weights (W_0_), then incubated in 5 mL SBF at 37 °C for different time periods. After incubation, samples were gently blotted to remove surface liquid, then weighed again to determine the final weight (W_t_). Relative mass change (%) was calculated as Equation (2):

$$Relative\;mass(\%)=\frac{\left(W_{t}-W_{0}\right)}{W_{0}}\times 100$$

In parallel, the pH of the SBF solution was monitored at the same time points to evaluate changes associated with scaffold degradation.

### 3.6. SBF Exposure Assay of the Scaffolds

The elemental composition of the scaffolds, with emphasis on calcium and phosphorus, was evaluated by X-ray fluorescence spectrometry (XRF) using a Mini-X2 spectrometer (Amptek Inc., Bedford, MA, USA). The analysis was carried out before and after 14 days of immersion of the scaffolds in SBF at 37 °C, to identify any possible changes in chemical composition resulting from degradation and/or surface mineralization. The measurements were taken at an operating condition of 30 kV and 50 µA. SEM was used to observe the surface morphology of these scaffolds before and after SBF testing. This approach allows morphological and elemental changes associated with mineralized layer formation under conditions close to a physiological microenvironment.

### 3.7. Cell Culture

Pre-osteoblastic MC3T3-E1 cells, established from the calvaria of C57BL/6 mice and obtained from the Rio de Janeiro Cell Bank (BCRJ, Rio de Janeiro, Brazil), as well as CHO-K1 cells derived from adult Chinese hamster ovary (*Cricetulus griseus*), obtained from the Rio de Janeiro Cell Bank (BCRJ, Rio de Janeiro, Brazil), were used in this study. MC3T3-E1 cells were employed for cytocompatibility, cell proliferation, nitric oxide (NO) production, oxidative stress, cell adhesion, and migration assays, whereas CHO-K1 cells were selected for the micronucleus assay due to their high sensitivity to genotoxic and clastogenic agents. All experiments were conducted in accordance with the OECD Guidance Document on Good In Vitro Method Practices [82], and ISO 10993-5:2009 guidelines for the biological evaluation of medical devices [56]. MC3T3-E1 cells were cultured in α-MEM medium (Vitrocell Embriolife, Campinas, Brazil), while CHO-K1 cells were maintained in F-12K medium (Vitrocell Embriolife, Campinas, Brazil) supplemented with 10% fetal bovine serum (FBS). Cell cultures were maintained at 37 °C in a humidified atmosphere containing 5% CO_2_ until approximately 80% confluence, with routine passaging performed as required to preserve cell viability and morphology.

### 3.8. Metabolic Activity and Cell Morphology

The effects of different composite compositions on the metabolic activity of MC3T3-E1 cells, as an indicator of cytotoxicity, were investigated using a fluorometric resazurin-based assay (Sigma-Aldrich, St. Louis, MO, USA). Cells were seeded in complete culture medium at a density of 1 × 10^4^ cells per well in 96-well plates (Corning Incorporated, Corning, NY, USA) and incubated for 24 h to allow cell adhesion. Subsequently, the cultures were exposed to conditioned media obtained from scaffolds fabricated with bHA or cHA (see Table 1). The assays were performed after 1, 3, 7, and 14 days of exposure. At each time point, cellular metabolic activity was determined by adding a sterile resazurin working solution (70 µM, prepared in 1× PBS) and incubating for 4 h at 37 °C in the dark to prevent photodegradation. Fluorescence was measured at excitation/emission wavelengths of 560/590 ± 10 nm using a GloMax^®^ Discover microplate reader (Promega Corporation, Madison, WI, USA). Fluorescence values were corrected for background and normalized to the negative control, which was set to 100% cell viability.

According to ISO 10993-5:2009, cell viability values ≥ 70% were considered non-cytotoxic; <50%, cytotoxic; and 50–70%, moderately cytotoxic [56]. All assays were performed in biological triplicate (*n* = 6), and the results are presented as mean ± standard deviation. In parallel with the assays, representative images of the cells for each condition tested were captured during the assay using an inverted optical microscope AXI0 (100×, Axio Observer, Carl Zeiss Microscopy GmbH, Oberkochen, Germany) equipped with a digital image acquisition system. The images aided in the qualitative evaluation of cell morphology.

### 3.9. Cell Proliferation

Cell proliferation was assessed by quantification of total DNA content using the PicoGreen^®^ assay. MC3T3-E1 cells (1 × 10^4^ cells per well) were seeded into 96-well culture plates and allowed to adhere under standard culture conditions. Experiments were conducted on days 1, 3, 7, and 14. At each time point, the conditioned culture medium was removed, wells were washed twice with PBS, and 100 µL of phosphate-buffered saline solution (PBS, Vitrocell Embriolife, Campinas, Brazil) was added. Cell lysis was achieved by a thermal shock procedure consisting of three freeze–thaw cycles (−80 °C and 25 °C), combined with mechanical disruption by pipetting. The resulting cell lysates were collected. In separate black 96-well plates, 200 µL of a freshly prepared working solution of 1× TE buffer (prepared with nuclease-free water; (Thermo Fisher Scientific, Waltham, MA, USA) containing PicoGreen^®^ dsDNA reagent was added to wells containing 10 µL of each sample or DNA standard. The plate was incubated in the dark for 5 min. Fluorescence was measured at excitation/emission wavelengths of 504/531 ± 10 nm using a GloMax^®^ Discover microplate reader (Promega Corporation, Madison, WI, USA). Fluorescence values were corrected for background signal, and DNA concentration was determined based on a calibration curve generated with known double-stranded DNA standards (nM), according to the manufacturer’s instructions for the PicoGreen^®^ dsDNA Quantitation Reagent (Promega Corporation, Madison, WI, USA). All experiments were performed in biological triplicate (*n* = 6), and results were expressed as mean ± standard deviation.

### 3.10. Evaluation of Intracellular Oxidative Stress

MC3T3-E1 cells (1 × 10^4^ cells/well) were plated in black 96-well culture plates (Corning Incorporated, Corning, NY, USA) and incubated for 24 h to allow cell adhesion. Subsequently, the cultures were exposed to conditioned media for 1, 3, 7, and 14 days. After the respective exposure periods, 2′,7′-dichlorodihydrofluorescein diacetate (H_2_DCFDA, 100 µM, Invitrogen, Thermo Fisher Scientific, Waltham, MA, USA) was added (100 µL), followed by a 30 min incubation in the dark. Fluorescence intensity was measured using a GloMax^®^ Discover microplate reader (Promega Corporation, Madison, WI, USA) at excitation and emission wavelengths of 485 and 530 nm, respectively. The emitted fluorescence was expressed as relative fluorescence units (RFUs) and normalized to the untreated control. All experiments were performed in biological triplicate (*n* = 9), and results were expressed as mean ± standard deviation.

### 3.11. Nitric Oxide Analysis

MC3T3-E1 osteoblast cells were seeded at a density of 1 × 10^4^ cells/well in 96-well plates (Corning Incorporated, Corning, NY, USA) and incubated for 24 h under standard conditions to allow adhesion. The study was performed after 1, 3, 7, and 14 days of incubation. At each specific time point, 100 µL of the culture supernatant was removed and added to another plate containing an equal amount of 100 µL of Griess reagent, prepared by combining equal volumes of Solution A (1% sulfanilamida (99% purity, Synth, Diadema, Brazil) in phosphoric acid (85% purity, Sigma-Aldrich, St. Louis, MO, USA)) and Solution B (0.1% N-(1-naphthyl)ethylenediamine dihydrochloride (98% purity, Sigma-Aldrich, St. Louis, MO, USA)). The reaction mixture was left to stand for 15 min at room temperature, protected from light, and then the absorbance was measured at 540 nm using a BioTek Instruments microplate spectrophotometer (Promega Corporation, Madison, WI, USA). Nitrite levels were used as an indirect measure of nitric oxide-derived reactive nitrogen species, which were quantified using a calibration curve constructed with known nitrite standards (nM), according to the manufacturer’s instructions for the modified Griess reagent kit (G4410, Sigma-Aldrich, St. Louis, MO, USA). All experiments were conducted in biological triplicate (*n* = 6), and results were expressed as mean ± standard deviation.

### 3.12. Cell Adhesion

MC3T3-E1 cells were seeded directly onto the surface of the scaffolds at 1 × 10^5^ cells per scaffold. The scaffolds were pre-wetted with complete culture medium for 24 h. The samples were incubated under standard culture conditions for an additional 24 h. Samples were incubated under standard culture conditions. Cell adhesion was evaluated after 24 h by confocal laser scanning microscopy using an SP8 AOBS Tandem Scanner System (Leica Microsystems GmbH, Wetzlar, Germany) based on fluorescent staining of the actin cytoskeleton with Alexa Fluor^TM^ 488-conjugated phalloidin (Invitrogen, Thermo Fisher Scientific, Waltham, MA, USA), and the nuclei with 4′,6-diamidino-2-phenylindole (DAPI; Invitrogen, Thermo Fisher Scientific, Waltham, MA, USA). Before image acquisition, the scaffolds were gently rinsed with PBS to remove non-adherent cells. Adherent cells were fixed with 4% paraformaldehyde (PFA, Synth, Diadema, Brazil) and subsequently stained with Alexa Fluor 488-conjugated phalloidin to visualize the actin cytoskeleton. Nuclear structures were stained using DAPI.

### 3.13. Cell Migration

Cell migration was evaluated using an indirect wound-healing (scratch) assay to assess the ability of the different composite compositions to promote or modulate the migratory behavior of MC3T3-E1 cells. Cells (1 × 10^5^ cells per well) were seeded in 12-well plates (Corning Incorporated, Corning, NY, USA) and cultured for 24 h under standard conditions until a confluent monolayer was formed. A straight and linear scratch was then created across the cell monolayer using a sterile 200 µL pipette tip, guided by a sterile ruler. Detached cells were gently removed by washing with PBS, and the medium was replaced with the respective conditioned media supplemented with 1% FBS to suppress cell proliferation and isolate migration-related effects. Wound closure was monitored immediately after scratching (0 h) and after 24 and 48 h of incubation using an inverted phase-contrast microscope AXI0 (Axio, 100×; Carl Zeiss Microscopy GmbH, Oberkochen, Germany). Representative images were acquired at each time point, and the wound area was quantified using ImageJ software (version 1.54p, National Institutes of Health, Bethesda, MD, USA). The percentage of wound closure was calculated according to the method described by Yue et al. [83], using the following Equation (3)

$$\%\;Wound\;closure=\frac{\left(A_{t=0h}-A_{t=\Delta h}\right)}{A_{t=0h}}\times 100$$

where $A_{t=0h}$ represents the wound area immediately after scratch formation, and $A_{t=\Delta h}$ corresponds to the wound area measured after 24 or 48 h of incubation with the respective treatments. All experiments were performed in biological triplicate (*n* = 3), and the results were expressed as mean ± standard deviation.

### 3.14. Extracellular Matrix Mineralization

MC3T3-E1 osteoblastic cells were seeded at a density of 1 × 10^5^ cells per well in 12-well plates (Corning Incorporated, Corning, NY, USA) and maintained under standard culture conditions for 24 h to allow cell adhesion. At the end of this period, the culture medium was replaced by the respective conditioned medium. Mineralization assays were performed after 7 and 14 days of incubation. Staining with Alizarin Red S (ARS, Sigma-Aldrich, St. Louis, MO, USA; pH 4.2) was used to assess extracellular matrix mineralization at each experimental time point. Cells were gently washed with PBS and then fixed in a 4% paraformaldehyde solution (PFA, Synth, Diadema, Brazil) for 15 min. Then, the wells were rinsed three times with PBS and incubated in 2% (*w*/*v*) ARS for 15 min, followed by washing with distilled water to remove excess dye. For quantification, the mineral-bound dye was solubilized with a 10% (*w*/*v*) cetylpyridinium chloride solution (Synth, Diadema, Brazil) which was added directly to each well. After 1 h of agitation, the resulting solution was transferred to a new 96-well plate (Corning Incorporated, Corning, NY, USA), and absorbance was read at 570 nm in a Biotek Instrument microplate spectrophotometer (Promega Corporation, Madison, WI, USA). The negative control received only basal medium, while osteogenic medium (α-MEM medium supplemented with 10% (*v*/*v*) FBS, 1% (*w*/*v*; Sigma-Aldrich, St. Louis, MO, USA) β-glycerophosphate, 1% (*w*/*v*; Sigma-Aldrich, St. Louis, MO, USA) trisodium salt of 2-phospho-L-ascorbic acid, and 0.1% (*w*/*v*; Sigma-Aldrich, St. Louis, MO, USA) dexamethasone) was used as the positive control.

### 3.15. Genotoxicity

The micronucleus assay was conducted by indirect contact using the CHO-K1 cell line as per the OECD Test Guideline 487 [70]. Initially, 0.5 × 10^6^ cells were seeded in 6-well plates (Corning Incorporated, Corning, NY, USA) and cultured for 24 h under standard conditions to allow cell adhesion. Different conditioned media with cHA or bHA with different concentrations were then used to treat these cells, allowing the genotoxic effects mediated by soluble components released from these scaffolds to be studied. After 24 h of exposure, the treatments were replaced with fresh F-12K medium containing cytochalasin B (4.5 μg/mL, Sigma-Aldrich, St. Louis, MO, USA), and the cells were incubated for an additional 24 h to block cytokinesis. The media were then aspirated, and the cells were washed twice with PBS, then trypsinized and harvested at 1500 rpm for 5 min. The pellet was resuspended in a cold hypotonic solution 0.075 M made with potassium chloride (KCl, 99% purity, Synth, Diadema, Brazil) at 4 °C for 20 min, then fixed twice with methanol/acetic acid (3:1, *v*/*v*; Sigma-Aldrich, St. Louis, MO, USA) for 15 min each time. The resulting cells were dropped onto pre-cleaned microscope slides, air dried, and then stained with a rapid panoptic kit (Newprov, Pinhais, PR, Brazil). The slides were analyzed using a Nikon optical microscope at 400× magnification and scored for micronuclei in at least 1000 binucleated cells per sample. Basal medium served as the negative control, while camptothecin (1 μM; HPLC grade; Sigma-Aldrich, St. Louis, MO, USA) was used as the positive control. All experiments were run in biological triplicate with six samples per treatment (*n* = 6). Results are expressed as mean ± standard deviation.

### 3.16. Statistical Analysis

Statistical analyses were performed using GraphPad Prism software (version 7.0). Data distribution was initially assessed through descriptive statistics and frequency histograms. Normality was then evaluated using the Shapiro-Wilk test. The results were expressed as mean ± standard deviation (SD) for parametric data. The differences between groups were tested using one-way analysis of variance (ANOVA) followed by Dunnett’s post hoc test to determine which group differed significantly from the control group. A *p*-value of <0.05 was considered statistically significant.

## 4. Conclusions

This study successfully established a protocol for the fabrication of composite scaffolds by 3D printing, using calcium alginate as a polymeric matrix reinforced with hydroxyapatite. Comparative analysis between cHA and bHA revealed that the bHA derived from croaker fish bones conferred superior crystallinity, likely due to the thermal extraction treatment. This characteristic influences the structural and biological behavior of the 3D-printed scaffolds. Regarding processing, all compositions maintained the expected rheological properties. However, the printed scaffolds containing 20% hydroxyapatite exhibited a visibly denser and less porous architecture. SEM analysis confirmed these morphological features, which improved E_c_ at the expense of σ_u_ and permeability, a direct consequence of scaffold densification. However, the formulation with 10% bHA showed a more favorable balance, maintaining sufficient porosity for metabolic exchange and promoting a significant increase in phosphorus deposition during immersion in SBF, indicating high bioactivity and greater apatite nucleation capacity.

In vitro results demonstrated higher bioactivity for the bHA10 samples, as these scaffolds proved to be non-toxic and non-genotoxic, sustaining metabolic activities well above the established biocompatibility thresholds. The bHA10 scaffold also strongly influenced cell behavior, promoting higher cell adhesion, migration, and proliferation in MC3T3-E1 pre-osteoblasts compared with the other groups. The absence of increased ROS and RNS, associated with the high mineralization observed by ARS staining after 14 days, confirms that bHA not only mimics the natural mineral phase of bone but also actively promotes osteogenic differentiation. This work demonstrates that recycling byproducts from the fishing industry into high-value-added biomaterials constitutes a viable and efficient strategy. The bHA10 scaffold stands out as a strong candidate for applications in bone tissue engineering, offering a unique combination of printability, biocompatibility, and pro-osteogenic signaling that surpasses conventional synthetic alternatives. Although the mechanical properties indicate that these scaffolds are not intended for high-load-bearing applications, the bHA10 scaffold stands out as a promising candidate for low-load bone tissue engineering applications, offering a unique combination of printability, biocompatibility, bioactivity, and pro-osteogenic signaling that surpasses conventional synthetic alternatives.

## Figures and Tables

**Table 1:** Experimental groups and their respective mass ratios.

Code	Mass Ratio in the Final Mixture (wt%)	
	NaAlg	HA
CaAlg	100	0
cHA10	100	10
cHA15	100	15
cHA20	100	20
bHA10	100	10
bHA15	100	15
bHA20	100	20

**Table 2:** Scaffold printing parameters.

Printing Parameters	
Layer height	0.6 mm
Infill density	70%
Infill pattern	gyroid
Infill angle	90°
Number of skirt loops	single-turn
Skirt distance	6 mm
Skirt height	1 layer
Infill speed	1.5 mm/s
Support material infill speed	1.5 mm/s
Travel speed	20 mm/s
Maximum printing speed	1.5 mm/s
Extrusion width	1 mm
First layer extrusion width	1 mm
Infill extrusion width	1 mm
Perimeter extrusion width	1 mm

## Data Availability

The data supporting the findings of this study are available within this article and its Supplementary Materials. Further inquiries may be directed to the corresponding author, and additional data will be made available upon reasonable request.

## Supplementary Materials

The following supporting information can be downloaded at: https://www.mdpi.com/article/10.3390/md24080260/s1, Figure S1: Heatmap-based visualization of relative mass change, presenting pairwise comparisons with the CaAlg (control) at each experimental time point; Figure S2: (**A**) Temporal variation of SBF pH after scaffold immersion over the same experimental pe-riods. (**B**) Heatmap-based visualization of pH variation, showing comparisons with the SBF (control) at each corresponding time point; Figure S3: Representative micrographs of the indirect cell viability assay obtained on days 1, 3, 7, and 14; Figure S4: 3D model used for the fabrication of cHA and bHA scaffolds; Table S1: Chemical composition of Simulated Body Fluid (SBF) prepared at pH 7.4 (1 L).

## Abbreviations

The following abbreviations are used in this manuscript:

SEM	Scanning electron microscopy
EDS	Energy dispersive spectroscopy
XRF	X-ray fluorescence
XRD	X-ray diffraction
HA	Hydroxyapatite
cHA	Commercial hydroxyapatite
bHA	Biogenic hydroxiapatite
G′	Storage modulus
G″	Loss modulus
LVR	Linear viscoelastic region
M block	Mannuronate
G-block	Guluronate
E_c_	Compressive modulus
σ_u_	Ultimate compressive strength
SBF	Simulated body fluid
NaOH	Sodium hydroxide
H_2_O_2_	Hydrogen peroxide
NaAlg	Sodium alginate
CaAlg	Calcium alginate
NO	Nitric oxide
FBS	Fetal bovine serum
CaCl_2_	Calcium chloride
ARS	Alizarin red S
KCl	Potassium chloride
ROS	Reactive oxygen species
H_2_DCFDA	2′,7′-dichlorodihydrofluorescein diacetate
RNS	Reactive nitrogen species
DAPI	4′,6-diamidino-2-phenylindole
SGC	Soluble guanylate cyclase

## References

[1] Shen Y., Huang X., Wu J., Lin X., Zhou X., Zhu Z., Pan X., Xu J., Qiao J., Zhang T. (2022). The Global Burden of Osteoporosis, Low Bone Mass, and Its Related Fracture in 204 Countries and Territories, 1990–2019. Front. Endocrinol. (Lausanne).

[2] Iliaens J., Onsea J., Hoekstra H., Nijs S., Peetermans W.E., Metsemakers W.-J. (2021). Fracture-Related Infection in Long Bone Fractures: A Comprehensive Analysis of the Economic Impact and Influence on Quality of Life. Injury.

[3] Yan J., Li F., Zhou J., Ding Y., Qin Q., Jin C. (2025). The Global Burden of Fractures and Its Underlying Etiologies: Results from and Further Analysis of the Global Burden of Disease Study 2021. Arch. Osteoporos..

[4] Fattahian H., Mansouri K., Mansouri N. (2019). Biomaterials, Substitutes, and Tissue Engineering in Bone Repair: Current and Future Concepts. Comp. Clin. Path..

[5] Grasser G.A., Nina D.G.N., Sousa K.S.J., Bonifacio M., Granito R.N., Moreira A.J., Rennó A.C.M., Longo E., Assis M. (2026). Bioactive chitosan scaffolds reinforced with hydroxyapatite and nickel tungstate for bone tissue engineering. Biomater. Sci..

[6] Wang Z., Sun Y., Li C. (2024). Advances in 3D Printing Technology for Preparing Bone Tissue Engineering Scaffolds from Biodegradable Materials. Front. Bioeng. Biotechnol..

[7] Beheshtizadeh N., Seraji A.A., Azadpour B., Rezvantalab S. (2025). The Stability and Self-Assembly of Tri-Calcium Silicate and Hydroxyapatite Scaffolds in Bone Tissue Engineering Applications. J. Biol. Eng..

[8] Sousa K.S.J., de Souza A., Assis M., Cruz M.A., Santo G.E., Nina D.G.N., Prado J.P.S., Moreira A.J., Granito R.N., Rennó A.C.M. (2026). Marine-Inspired Three-Dimensional Printed Biosilica-Spongin Scaffolds as Promoters of Osteogenic Differentiation and Gene Expression. ACS Appl. Bio Mater..

[9] Aslam Khan M.U., Aslam M.A., Bin Abdullah M.F., Stojanović G.M. (2024). Current Perspectives of Protein in Bone Tissue Engineering: Bone Structure, Ideal Scaffolds, Fabrication Techniques, Applications, Scopes, and Future Advances. ACS Appl. Bio Mater..

[10] Xu C., Liu Z., Chen X., Gao Y., Wang W., Zhuang X., Zhang H., Dong X. (2024). Bone Tissue Engineering Scaffold Materials: Fundamentals, Advances, and Challenges. Chin. Chem. Lett..

[11] Rodríguez-González R., Delgado L.M., Pérez R.A. (2025). Achievements in 3D Printing of Silica-Based Materials for Bone Tissue Engineering. Colloids Surf. B Biointerfaces.

[12] Liu Y., Luo D., Wang T. (2016). Hierarchical Structures of Bone and Bioinspired Bone Tissue Engineering. Small.

[13] Rahman S.U. (2020). Hydroxyapatite and Tissue Engineering. Handbook of Ionic Substituted Hydroxyapatites.

[14] Liu W., Cheong N., He Z., Zhang T. (2025). Application of Hydroxyapatite Composites in Bone Tissue Engineering: A Review. J. Funct. Biomater..

[15] Farazin A., Darghiasi S.F. (2025). Hydroxyapatite-based Nanocomposite Scaffolds for Bone Regeneration. Int. J. Appl. Ceram. Technol..

[16] Owen G.R., Dard M., Larjava H. (2018). Hydoxyapatite/Beta-tricalcium Phosphate Biphasic Ceramics as Regenerative Material for the Repair of Complex Bone Defects. J. Biomed. Mater. Res. B Appl. Biomater..

[17] Zhou H., Lee J. (2011). Nanoscale Hydroxyapatite Particles for Bone Tissue Engineering. Acta Biomater..

[18] Danapriya S., Priya S.P.S., Prasadh S.H., Sathish J., Solomon Godwin Babu N.D. (2022). Extraction of Value-Added Products from Marine Resources. Russ. J. Mar. Biol..

[19] Okpe P.C., Folorunso O., Aigbodion V.S., Obayi C. (2024). Hydroxyapatite Synthesis and Characterization from Waste Animal Bones and Natural Sources for Biomedical Applications. J. Biomed. Mater. Res. B Appl. Biomater..

[20] de Souza A., de Almeida Cruz M., de Araújo T.A.T., Parisi J.R., do Vale G.C.A., Dos Santos Jorge Sousa K., Ribeiro D.A., Granito R.N., Renno A.C.M. (2022). Fish Collagen for Skin Wound Healing: A Systematic Review in Experimental Animal Studies. Cell Tissue Res..

[21] Granito R.N., Renno A.C., Yamamura H., Cruz de Almeida M., Luiz Menin Ruiz P., Araki Ribeiro D. (2018). Hydroxyapatite from Fish for Bone Tissue Engineering: A Promising Approach. Int. J. Mol. Cell. Med..

[22] Seikh Z., De B., Ghosh D., Saha S., Bera S., Mandal G., Sinha A. (2024). An Overview on the Utilization of Hydroxyapatite from Fish Wastes: A Transition from Waste to Wealth. J. Inst. Eng. (India) Ser. D.

[23] Xavier B.G., da Silva L.V., Alves F.C., Teixeira L.V., Santos V.E.N. (2024). Circular Bioeconomy Ecosystems: Hydroxyapatite from Fish Waste at Rio de Janeiro. Biofuels Bioprod. Biorefining.

[24] Shi P., Liu M., Fan F., Yu C., Lu W., Du M. (2018). Characterization of Natural Hydroxyapatite Originated from Fish Bone and Its Biocompatibility with Osteoblasts. Mater. Sci. Eng. C.

[25] Venkatesan J., Lowe B., Manivasagan P., Kang K.-H., Chalisserry E., Anil S., Kim D., Kim S.-K. (2015). Isolation and Characterization of Nano-Hydroxyapatite from Salmon Fish Bone. Materials.

[26] Boutinguiza M., Pou J., Comesaña R., Lusquiños F., de Carlos A., León B. (2012). Biological Hydroxyapatite Obtained from Fish Bones. Mater. Sci. Eng. C.

[27] Wardani S.C., Sujuti H., Mustamsir E., Hapsari D.N. (2020). Synthesis and Potential of Skipjack Tuna Bone Hydroxyapatite as Bone Tissue Engineering Biomaterial. J. Phys. Conf. Ser..

[28] Gnanasekaran R., Yuvaraj D., Muthu C.M.M., Ashwin R., Kaarthikeyan K., Kumar V.V., Ramalingam R.J., Al-Lohedan H., Reddy K. (2024). Extraction and Characterization of Biocompatible Hydroxyapatite (Hap) from Red Big Eye Fish Bone: Potential for Biomedical Applications and Reducing Biowastes. Sustain. Chem. Environ..

[29] Pal A., Paul S., Choudhury A.R., Balla V.K., Das M., Sinha A. (2017). Synthesis of Hydroxyapatite from Lates Calcarifer Fish Bone for Biomedical Applications. Mater. Lett..

[30] Muñoz F., Haidar Z.S., Puigdollers A., Guerra I., Padilla M.C., Ortega N., García M.J. (2024). A Novel Chilean Salmon Fish Backbone-Based NanoHydroxyApatite Functional Biomaterial for Potential Use in Bone Tissue Engineering. Front. Med. (Lausanne).

[31] Zhu Q., Ablikim Z., Chen T., Cai Q., Xia J., Jiang D., Wang S. (2017). The Preparation and Characterization of HA/β-TCP Biphasic Ceramics from Fish Bones. Ceram. Int..

[32] Nam P.V., Van Hoa N., Trung T.S. (2019). Properties of Hydroxyapatites Prepared from Different Fish Bones: A Comparative Study. Ceram. Int..

[33] Tanaka Y., Iwasaki T., Katayama K., Hojo J., Yamashita K. (2010). Effect of Ionic Polarization on Crystal Structure of Hydroxyapatite Ceramic with Hydroxide Nonstoichiometry. J. Jpn. Soc. Powder Powder Metall..

[34] Landi E., Celotti G., Logroscino G., Tampieri A. (2003). Carbonated Hydroxyapatite as Bone Substitute. J. Eur. Ceram. Soc..

[35] Machado T.R., Sczancoski J.C., Beltrán-Mir H., Nogueira I.C., Li M.S., Andrés J., Cordoncillo E., Longo E. (2017). A Novel Approach to Obtain Highly Intense Self-Activated Photoluminescence Emissions in Hydroxyapatite Nanoparticles. J. Solid State Chem..

[36] Min K.H., Kim D.H., Kim K.H., Seo J.-H., Pack S.P. (2024). Biomimetic Scaffolds of Calcium-Based Materials for Bone Regeneration. Biomimetics.

[37] House A., Kuna A., Hastings D., Rodriguez N., Schoenitz M., Dreizin E.L., Guvendiren M. (2023). Effect of Particle Shape on Rheology and Printability of Highly Filled Reactive Inks for Direct Ink Writing. Prog. Addit. Manuf..

[38] Lee C.P., Takahashi M., Arai S., Lee C.-L.K., Hashimoto M. (2021). 3D Printing of Okara Ink: The Effect of Particle Size on the Printability. ACS Food Sci. Technol..

[39] Ramos-Souza C., Fernandes A.S., Mazzo T.M., Perrechil F., De Rosso V.V. (2025). Fine-Tuning Carotenoid-Enriched Bigel Formulations: Exploring the Influence of Oleogel: Hydrogel Ratio on Physicochemical Properties and 3D Food Printing. Food Hydrocoll..

[40] Helmiyati, Aprilliza M. (2017). Characterization and Properties of Sodium Alginate from Brown Algae Used as an Ecofriendly Superabsorbent. IOP Conf. Ser. Mater. Sci. Eng..

[41] Cheng W., Zhang Q., Xue Y., Wang Y., Zhou X., Li Z., Li Q. (2021). Facile Synthesis of Alginate-based Calcium Tungstate Composite: A Thermally Stable Blue Emitting Phosphor. J. Appl. Polym. Sci..

[42] Assis M., Cano-Vicent A., Tuñon-Molina A., Benzi-Chumachenco R.R., Andrés J., Serrano-Aroca A. (2024). Calcium Alginate Films Loaded with Copper-Molybdenum Oxide Nanoparticles for Antimicrobial Applications. J. Environ. Chem. Eng..

[43] Hossain S., Ahmed S. (2023). FTIR Spectrum Analysis to Predict the Crystalline and Amorphous Phases of Hydroxyapatite: A Comparison of Vibrational Motion to Reflection. RSC Adv..

[44] do Espirito Santo G., de Souza A., Amaral G.O., Alqualo A.S., Sousa K.d.S.J., Viegas B.L.M., Prado J.P.d.S., dos Santos F.V., Correa D.S., Granito R.N. (2025). Development and Comparison of Two 3D-Printed Scaffolds of Biosilica from Marine Sponges for Bone Tissue Engineering. ACS Omega.

[45] Picado-Tejero D., Mendoza-Cerezo L., Rodríguez-Rego J.M., Carrasco-Amador J.P., Marcos-Romero A.C. (2025). Recent Advances in 3D Bioprinting of Porous Scaffolds for Tissue Engineering: A Narrative and Critical Review. J. Funct. Biomater..

[46] Wu S., Liu X., Yeung K.W.K., Liu C., Yang X. (2014). Biomimetic Porous Scaffolds for Bone Tissue Engineering. Mater. Sci. Eng. R Rep..

[47] Kane R.J., Roeder R.K. (2012). Effects of Hydroxyapatite Reinforcement on the Architecture and Mechanical Properties of Freeze-Dried Collagen Scaffolds. J. Mech. Behav. Biomed. Mater..

[48] Zhang H., Zhou L., Zhang W. (2014). Control of Scaffold Degradation in Tissue Engineering: A Review. Tissue Eng. Part B Rev..

[49] Dridi A., Riahi K.Z., Somrani S. (2021). Mechanism of Apatite Formation on a Poorly Crystallized Calcium Phosphate in a Simulated Body Fluid (SBF) at 37 °C. J. Phys. Chem. Solids.

[50] Cao J., Lian R., Jiang X., Liu X. (2020). Formation of Porous Apatite Layer after Immersion in SBF of Fluorine-Hydroxyapatite Coatings by Pulsed Laser Deposition Improved in Vitro Cell Proliferation. ACS Appl. Bio Mater..

[51] Bajpai S.K., Sharma S. (2004). Investigation of Swelling/Degradation Behaviour of Alginate Beads Crosslinked with Ca2+ and Ba2+ Ions. React. Funct. Polym..

[52] dos Santos Jorge Sousa K., de Souza A., de Almeida Cruz M., de Lima L.E., do Espirito Santo G., Amaral G.O., Granito R.N., Renno A.C. (2024). 3D Printed Scaffolds of Biosilica and Spongin from Marine Sponges: Analysis of Genotoxicity and Cytotoxicity for Bone Tissue Repair. Bioprocess Biosyst. Eng..

[53] Zamani Y., Mohammadi J., Amoabediny G., Visscher D.O., Helder M.N., Zandieh-Doulabi B., Klein-Nulend J. (2018). Enhanced Osteogenic Activity by MC3T3-E1 Pre-Osteoblasts on Chemically Surface-Modified Poly(*ε*-Caprolactone) 3D-Printed Scaffolds Compared to RGD Immobilized Scaffolds. Biomed. Mater..

[54] Han J., Park S., Kim J.E., Park B., Hong Y., Lim J.W., Jeong S., Son H., Kim H.B., Seonwoo H. (2023). Development of a Scaffold-on-a-Chip Platform to Evaluate Cell Infiltration and Osteogenesis on the 3D-Printed Scaffold for Bone Regeneration. ACS Biomater. Sci. Eng..

[55] Rampersad S.N. (2012). Multiple Applications of Alamar Blue as an Indicator of Metabolic Function and Cellular Health in Cell Viability Bioassays. Sensors.

[56] (2009). Biological Evaluation of Medical Devices—Part 5: Tests for In Vitro Cytotoxicity.

[57] Prado J.P.d.S., Yamamura H., Magri A.M.P., Ruiz P.L.M., Prado J.L.d.S., Rennó A.C.M., Ribeiro D.A., Granito R.N. (2021). In Vitro and in Vivo Biological Performance of Hydroxyapatite from Fish Waste. J. Mater. Sci. Mater. Med..

[58] DeWane G., Salvi A.M., DeMali K.A. (2021). Fueling the Cytoskeleton—Links between Cell Metabolism and Actin Remodeling. J. Cell Sci..

[59] Li X., de Assis Souza R., Heinemann M. (2025). The Rate of Glucose Metabolism Sets the Cell Morphology across Yeast Strains and Species. Curr. Biol..

[60] Tatapudy S., Aloisio F., Barber D., Nystul T. (2017). Cell Fate Decisions: Emerging Roles for Metabolic Signals and Cell Morphology. EMBO Rep..

[61] Zhan H., Pal D.S., Borleis J., Deng Y., Long Y., Janetopoulos C., Huang C.-H., Devreotes P.N. (2025). Self-Organizing Glycolytic Waves Tune Cellular Metabolic States and Fuel Cancer Progression. Nat. Commun..

[62] Bonifacio M., Santi Martignago C.C., Souza D.C.S., Garcia-Motta H., Souza-Silva L.C., Soares-Silva B., Sousa K.S.J., Rosso A.P., Lago J.H.G., Ribeiro A.M. (2025). Chitosan Hydrogels Enriched with Biocompounds Extracted from Marine Sponges: Potential to Modulate the Inflammatory Process in an In Vitro Study. ACS Omega.

[63] Mondal S., Hoang G., Manivasagan P., Moorthy M.S., Kim H.H., Vy Phan T.T., Oh J. (2019). Comparative Characterization of Biogenic and Chemical Synthesized Hydroxyapatite Biomaterials for Potential Biomedical Application. Mater. Chem. Phys..

[64] Fang Z., Wang Y., Feng Q., Kienzle A., Müller W.E.G. (2014). Hierarchical Structure and Cytocompatibility of Fish Scales from Carassius Auratus. Mater. Sci. Eng. C.

[65] Mouthuy P.-A., Snelling S.J.B., Dakin S.G., Milković L., Gašparović A.Č., Carr A.J., Žarković N. (2016). Biocompatibility of Implantable Materials: An Oxidative Stress Viewpoint. Biomaterials.

[66] Nascimento M.H.M., Pelegrino M.T., Pieretti J.C., Seabra A.B. (2020). How Can Nitric Oxide Help Osteogenesis?. AIMS Mol. Sci..

[67] Lee J., Kim D., Park S., Baek S., Jung J., Kim T., Han D.K. (2023). Nitric Oxide-Releasing Bioinspired Scaffold for Exquisite Regeneration of Osteoporotic Bone via Regulation of Homeostasis. Adv. Sci..

[68] Shafiq M., Chen Y., Hashim R., He C., Mo X., Zhou X. (2021). Reactive Oxygen Species-Based Biomaterials for Regenerative Medicine and Tissue Engineering Applications. Front. Bioeng. Biotechnol..

[69] Liang L., Li X., Liang H., Zhang J., Lu Q., Zhou G., Tang J., Li X. (2025). Navigating Oxidative Stress in Oral Bone Regeneration: Mechanisms and Reactive Oxygen Species-Regulating Biomaterial Strategies. Regen. Biomater..

[70] Assis M., de Souza A., dos Santos Jorge Sousa K., Nina D.G.N., Bonfacio M., Granito R.N., Rennó A.C.M. (2025). Deciphering the Toxicity of Metal Tungstates and Molybdates: Effects on L929 Cell Metabolic Activity, Oxidative Stress, and Genotoxicity. J. Appl. Toxicol..

[71] Pon-On W., Suntornsaratoon P., Charoenphandhu N., Thongbunchoo J., Krishnamra N., Tang I.M. (2016). Hydroxyapatite from Fish Scale for Potential Use as Bone Scaffold or Regenerative Material. Mater. Sci. Eng. C.

[72] Baek J.W., Kim K.S., Park H., Kim B.-S. (2022). Marine Plankton Exoskeletone-Derived Hydroxyapatite/Polycaprolactone Composite 3D Scaffold for Bone Tissue Engineering. Biomater. Sci..

[73] Fan Y., Yao J., Liu W., Wang L., Yang J., Zheng X., Hui J., Fan D. (2026). 3D Printed Composite Scaffold Accelerates Bone Regeneration by Modulating Immunity and Promoting Angiogenesis. J. Mater. Sci. Technol..

[74] Sistani S., Asgharzade S., Arab S., Bahraminasab M., Soltani-Fard E. (2025). Fabrication and Evaluation of a Host-Guest Polylactic Acid/Gelatin-Hydroxyapatite-Blueberry Scaffold for Bone Regeneration. J. Orthop. Surg. Res..

[75] Bernar A., Gebetsberger J.V., Bauer M., Streif W., Schirmer M. (2022). Optimization of the Alizarin Red S Assay by Enhancing Mineralization of Osteoblasts. Int. J. Mol. Sci..

[76] Jaita P., Randorn C., Watcharapasorn A., Jarupoom P. (2024). In Vitro Bioactivity, Mechanical, and Cell Interaction of Sodium Chloride-Added Calcium Sulfate–Hydroxyapatite Composite Bone Cements. RSC Adv..

[77] Yousefi M., Maffulli N., Bahraminasab M., Arab S., Alizadeh A., Ghanbari A., Talebi A., Jafari Sorkhdehi M.M. (2025). Critical-Size Bone Defect Repair with Three Types of Nano-Hydroxyapatite Scaffolds: An in Vivo Study. BioImpacts.

[78] de Souza A.M., Dantas M.R.d.N., Secundo E.L., Silva E.d.C., Silva P.F., Moreira S.M.G., de Medeiros S.R.B. (2024). Are Hydroxyapatite-Based Biomaterials Free of Genotoxicity? A Systematic Review. Chemosphere.

[79] Yamamura H., da Silva V.H.P., Ruiz P.L.M., Ussui V., Lazar D.R.R., Renno A.C.M., Ribeiro D.A. (2018). Physico-Chemical Characterization and Biocompatibility of Hydroxyapatite Derived from Fish Waste. J. Mech. Behav. Biomed. Mater..

[80] Kido H.W., Ribeiro D.A., de Oliveira P., Parizotto N.A., Camilo C.C., Fortulan C.A., Marcantonio E., da Silva V.H.P., Muniz Renno A.C. (2014). Biocompatibility of a Porous Alumina Ceramic Scaffold Coated with Hydroxyapatite and Bioglass. J. Biomed. Mater. Res. A.

[81] Castro M.S., Martins I.M., Hanazaki N. (2016). Trophic Relationships between People and Resources: Fish Consumption in an Artisanal Fishers Neighborhood in Southern Brazil. Ethnobiol. Conserv..

[82] (2016). Test No. 487.

[83] Yue P.Y.K., Leung E.P.Y., Mak N.K., Wong R.N.S. (2010). A Simplified Method for Quantifying Cell Migration/Wound Healing in 96-Well Plates. SLAS Discov..

