# Supplementary material for: Croaker Fish Bone-Derived Hydroxyapatite as a Sustainable Source for 3D-Printed Scaffolds for Bone Regeneration
Source: Mar Drugs. 2026 Jul 26;24(8):260. doi: 10.3390/md24080260 (PMC13514607; doi:10.3390/md24080260)
Supplement: Supplementary file 1 [file marinedrugs-24-00260-s001.zip › marinedrugs-4422776-supplementary.pdf]

Article

Croaker Fish Bone-Derived Hydroxyapatite as a Sustainable Source for 3D Printed Scaffolds for Bone Regeneration

Diana Gabriela Nina Nina [1], Giovanna de Amorim Grasser [2], Amanda Sardeli Alqualo [1], João Paulo dos Santos Prado [1], Eliandra de Sousa Trichês [3], Elson Longo [2], Ana Cláudia Muniz Rennó [1], Anna Rafaela Cavalcante Braga [1,4], Marcelo Assis [5,*] and Renata Neves Granito [1,*]

[1] Department of Biosciences, Universidade Federal de São Paulo (UNIFESP), Santos 11015-020, Brazil; dninal@unifesp.br (DGNN); amanda.assaf@unifesp.br (ASA); paulo.prado@unifesp.br (JPdSP); a.renno@unifesp.br (ACMR); anna.braga@unifesp.br (ARCB)

[2] Center for the Development of Functional Materials (CDMF), Universidade Federal de São Carlos (UFSCar), São Carlos 13565-905, Brazil; giovanna.grasser@estudante.ufscar.br (GdAG); elson.liec@gmail.com (EL)

[3] Institute of Science and Technology, Universidade Federal de São Paulo (UNIFESP), São José dos Campos 12231-280, Brazil; eliandra.sousa@unifesp.br (EST)

[4] Department of Chemical Engineering, Universidade Federal de São Paulo (UNIFESP), Diadema 09972-270, Brazil

[5] Institute for Advanced Marine Studies (IEAMar), São Paulo State University (UNESP), São Vicente 11350-011, Brazil

* Correspondence: m.assis1@unesp.br (MA); rn.granito@unifesp.br (RNG); Tel.: +55-1699798749 (MA); +55-13981082406 (RNG)

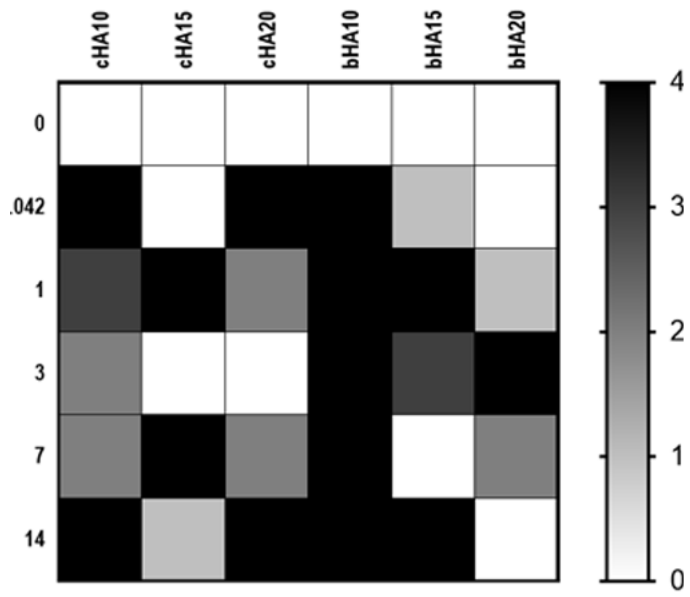

Figure S1. A) Heatmap-based visualization of relative mass change, presenting pairwise comparisons with the CaAlg (control) at each experimental time point. Differences were considered statistically significant when $p < 0.05$. In the heatmaps, statistical significance is encoded by intensity levels as follows: 0 = no statistical significance; 1 = *; 2 = **; 3 = ***; and 4 = ****.

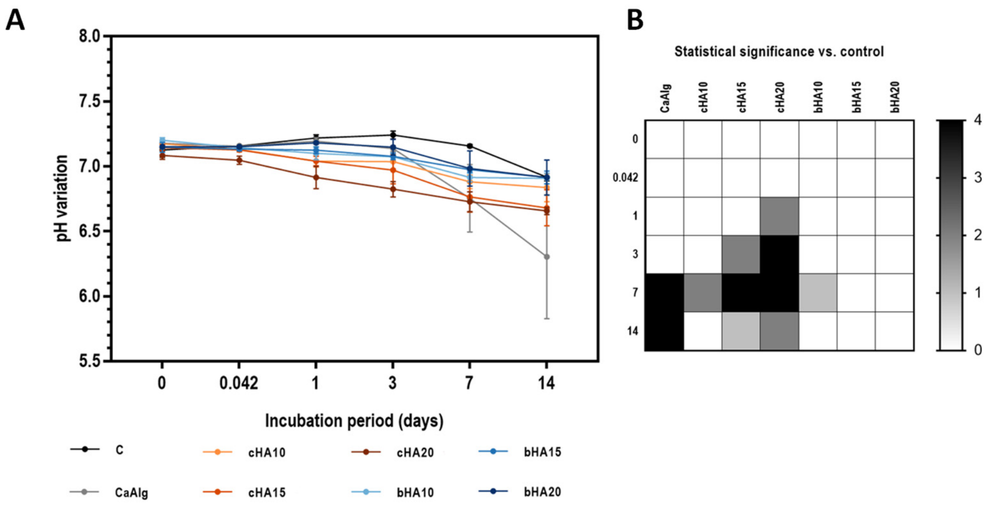

Figure S2. A) Temporal variation of SBF pH after scaffold immersion over the same experimental periods. **B)** Heatmap-based visualization of pH variation, showing comparisons with the SBF (control) at each corresponding time point. All data are presented as mean ± standard deviation. Statistical analyses were performed using one-way analysis of variance (ANOVA), followed by a post hoc test. Differences were considered statistically significant when $p < 0.05$. In the heatmaps, statistical significance is encoded by intensity levels as follows: 0 = no statistical significance; 1 = *; 2 = **; 3 = ***; and 4 = ****.

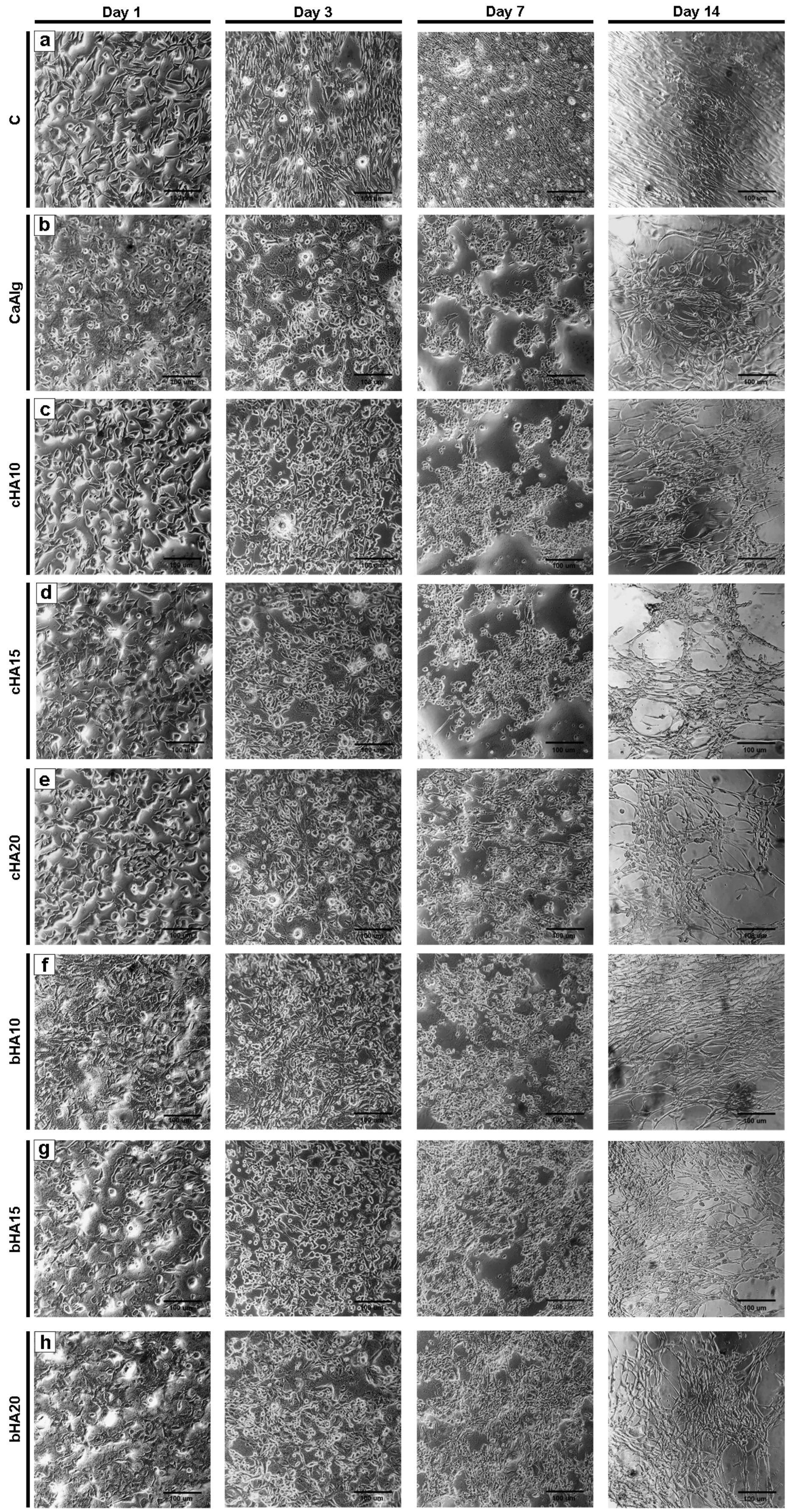

Figure S3. Representative micrographs of the indirect cell viability assay obtained on days 1, 3, 7, and 14.

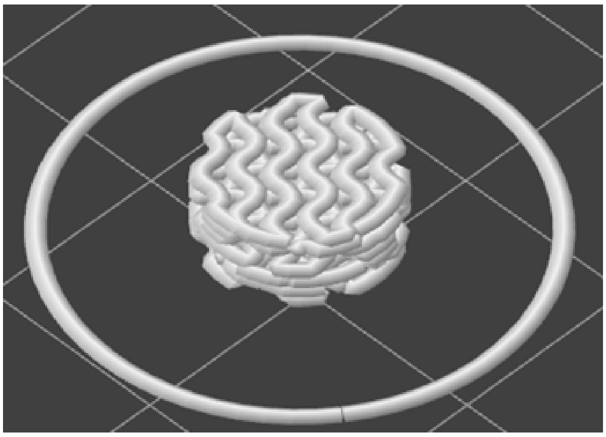

Figure S4. 3D model used for the fabrication of cHA and bHA scaffolds.

Table S1. Chemical composition of Simulated Body Fluid (SBF) prepared at pH 7.4 (1 L)

Chemical compound	Chemical formula	Amount (g/l)
Sodium chloride	$NaCl$	7.996
Sodium bicarbonate	$NaHCO_3$	0.350
Potassium chloride	KCl	0.224
Dipotassium phosphate trihydrate	$K_2HPO_4 \cdot 3H_3O$	0.228
Magnesium chloride hexahydrate	$MgCl_2 \cdot 6H_2O$	0.305
Calcium chloride	$CaCl_2$	0.278
Sodium sulfate	Na_2SO_4	0.071
Tris(hydroxymethyl)amino-methane (TRIS)	$(CH_2OH)_3CNH_2$	6.057
Hydrochloric acid (pH adjust-ment)	HCl 1 M	q.s. to pH 7.4
